# Geographical distribution of ixodid ticks and tick-borne pathogens of domestic animals in Ethiopia: a systematic review

**DOI:** 10.1186/s13071-022-05221-x

**Published:** 2022-03-28

**Authors:** Tamirat Kaba

**Affiliations:** Department of Veterinary and Animal Science, College of Agricultural Science, Arba Minch University, Arba Minch, Ethiopia

**Keywords:** Domestic animals, Ethiopia, Geographical distribution, Ixodid ticks, Tick-borne pathogens

## Abstract

**Background:**

In Ethiopia, ixodid ticks and associated tick-borne pathogens (TBPs) are of great importance from both a veterinary and public health point of view. This review aimed at compiling available published data on the distribution of ixodid tick species and TBPs in the country.

**Methods:**

A standard review approach was employed using the Preferred Reporting Items for Systematic Reviews and Meta-Analyses (PRISMA) guideline. Published peer-reviewed articles and theses/dissertations reporting on ixodid ticks and TBPs in Ethiopia were searched using different keywords in many electronic databases including PubMed, Scopus, Web of Science, Google Scholar, African Journals OnLine, and institutional repositories. Articles were screened based on inclusion and exclusion criteria using the PRISMA flowchart. Data were retrieved from eligible articles and recorded in a preformed data record sheet. Descriptive statistics were employed to present data using graphs. QGIS (Quantum GIS) software version 3.4.5 was used to show the distribution of ixodid tick species and TBPs.

**Results:**

Overall, 35 articles that met the inclusion criteria were included in this review. Of these, 24 articles report only on ixodid ticks of domestic animals, six articles report only on TBPs in livestock or ticks, and five articles report on both ticks and TBPs in either animals or ticks. Of these studies, 54% were in the Oromia region, while only 3% of studies were in the Benishangul-Gumuz region. The Gambela region lacked studies on ticks and TBPs. At least 19 ixodid tick species have been recorded from different domestic animals including cattle, small ruminants, donkeys, horses, camels, dogs, and cats. Morphological characterization appears to be the sole method of tick species identification in the country. The distribution and abundance of specific tick species depend on geographical locations and agroecological factors. Sixteen molecularly confirmed TBPs have been identified in animal and tick tissue using molecular methods from only four administrative regions, despite the wide distribution of ticks. Among TBPs, five *Anaplasma*, two *Ehrlichia*, two *Rickettsia*, five *Theileria*, two *Babesia*, and one *Coxiella* species are the major pathogens in both livestock and humans.

**Conclusions:**

Many ixodid ticks circulate in a wide geographical zone of Ethiopia. However, the limited reports on TBPs at the country level in general, and the absence of either tick or TBP reports around the border region with neighboring countries in particular, highlights the need for further study.

**Graphical Abstract:**

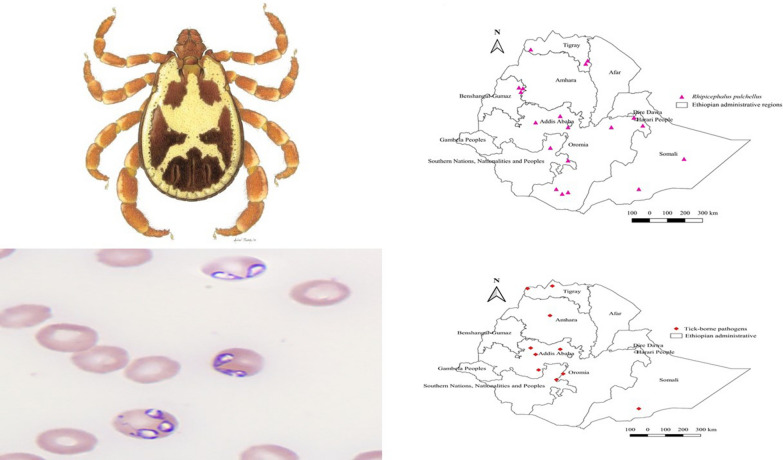

**Supplementary Information:**

The online version contains supplementary material available at 10.1186/s13071-022-05221-x.

## Background

In Ethiopia, the contribution of domestic animals to socioeconomic well-being remains substantial. Cattle, sheep, and goats contribute to the national gross domestic product (GDP) by providing meat and milk for local and international markets [1]. They are the source of raw materials for the leather industries in the country [2]. Sheep and goats are considered an emergency savings bank for immediate income for livestock farming communities [3]. Camels, which are kept mostly by the pastoral and agropastoral communities, serve as pack animals for transportation and as a source of milk [4]. Donkeys, mules, and horses provide pack and transportation services in both urban and rural settings [5, 6]. Dogs and cats are companion animals; in addition, dogs provide guard services [7]. Nevertheless, many factors hamper their optimal socioeconomic contribution, as parasitism in general, and ixodid ticks and associated pathogens in particular, poses substantial health threats to these animals.

Ixodid ticks are a group of ectoparasites whose life-cycle depends on the vertebrate host. The importance of ixodid ticks as ectoparasites is their habit of blood-feeding on domestic and wild animals and humans to complete their life-cycle. In the feeding process, the affected host may become anemic if the parasite burden is high [8] or may be exposed to secondary pathogens for which ticks are vectors. Besides blood-feeding, they pierce the coats of animals with their specialized mouthparts, and thereby cause wounds and hence scars on the coats of the animals. Consequently, the economic impact in terms of downgrading the quality of hide and skin in tanneries is significant [9].

Ticks are distributed worldwide, although the distribution of individual tick species varies according to climatic factors, such as temperature, humidity, altitude, and vegetation types. For instance, the tick *Rhipicephalus appendiculatus*, presumed to be prevalent in East African countries including in the highland region of Kenya, does not thrive in the border area of two countries where the unrestricted movement of livestock takes place. Unfavorable natural climatic barriers might have prevented the introduction of this tick to Ethiopia from neighboring Kenya [10]. This suggests that variations in the agroecology of different regions determine the biology and abundance of ticks.

Information concerning the distribution and abundance of ticks is of paramount importance, as it helps to predict the occurrence of tick-borne infections in animals so that control measures against ticks and associated infections can be set. This information can be used as a criterion during the morphological identification of tick fauna by inexperienced researchers [11]. Moreover, many tick-borne pathogens (TBPs) can be well diagnosed based on the availability of the specific tick distribution data. However, in the absence of such information, TBPs are misdiagnosed or wrongly reported. For example, the pathogen *Babesia bovis*, which causes bovine babesiosis in several regions, was wrongly reported from the southwestern part of Ethiopia, where neither of its vectors *Rhipicephalus* (Boophilus) *microplus* nor *Rhipicephalus* (Boophilus) *annulatus* was reported [12]. A letter to the editor was published concerning this misleading report from the southwestern region of the country [13]. Furthermore, other researchers [14] confirmed the absence of *B. bovis* in the area using molecular techniques. These examples illustrate the possibility of predicting TBPs without requiring expensive laboratory techniques when their vector distribution is known.

Indigenous breeds of livestock are resilient to ticks and TBPs, but genetically improved breeds, which contribute to sustainable food security, are highly susceptible [14, 15]. Various TBPs have been identified from animals [12, 15, 16] and from ticks themselves [17, 18]. However, the problem does not seem uniform across all agroecological zones, as several ticks known to be vectors for highly pathogenic agents for both improved and local breeds of animals have been found to select suitable agroecology for their survival and multiplication. This shows that TBPs often coincide with the distribution of their vectors. However, comprehensive information on the geographical distribution of specific tick species in the country is lacking, except for one historical study, which was reported 40 years ago [19]. Given the effect of climate change on the distribution of ticks over a period, reporting updated information is essential.

Therefore, this literature review is designed to present local secondary data on the distribution and abundance of ixodid tick species infesting various domestic animals in Ethiopia. It also demonstrates available evidence on TBP distribution in the country. This information is of paramount importance in designing control measures against ticks and TBPs in domestic animals.

## Methods

### Review protocol

The Preferred Reporting Items for Systematic reviews and Meta-Analyses (PRISMA) checklist and flowchart for data extraction and inclusion of eligible studies, respectively, were used for this study [20].

### Literature source and search methods

Literature databases including PubMed, Web of Science, Scopus, African Journals OnLine (AJOL), and Google Scholar were used to retrieve published articles on ixodid ticks and TBPs affecting domestic animals in Ethiopia. To maximize the search, the key terms “ixodid ticks of domestic animals,” “ticks of livestock,” “ectoparasite of domestic animals,” “ectoparasite of livestock,” ticks of ruminants,” “ticks of equines,” “ticks of cats and dogs,” ticks of the camel,” “tick and tick-borne disease of livestock,” AND “Ethiopia” were used. All works published from 2001 to October 30, 2020, were included. In addition, PhD/MSc theses that had not yet been published in peer-reviewed journals were searched from different institutional repository databases. A systematic screening of available pieces of literature was conducted by assessing the title, abstract, and detailed aspects of manuscripts.

### Inclusion and exclusion criteria

All articles indexed in the aforementioned databases that report on ixodid ticks alone or ixodid ticks in combination with other ectoparasites and TBPs in ticks and/or in domestic animals, including ruminants, camels, equines, and pets (dogs and cats) in Ethiopia were included. To investigate differences in the geographical and host abundance of tick species, only those articles that revealed the proportion of individual tick species in infested animals and those articles reporting TBPs in ticks/animals were included. However, studies in which ticks were not identified to species level and tick species proportions were not clearly reported were excluded. Mendeley Desktop version 1.15.3 was used to catalog the initial literature search results and manage citations.

### Data extraction

From eligible studies, the following data were extracted: the first author, year of publication, geographical location, administrative region of study, host species, proportion of tick species on animals, TBPs in ticks or animals, agroecology, and georeference of the study sites. The georeference data were obtained from the internet if it was not mentioned in the publication. The geographical locations of study sites were systematically classified into five categories, namely central, western, eastern, southern, and northern Ethiopia, based on the distance and direction of specific study districts from the capital city (Addis Ababa). Accordingly, studies conducted within a radius of 150 km from Addis Ababa were categorized as central. The designations southern, northern, eastern, and western were used for studies reported from areas more than 150 km from Addis Ababa in their respective directions. The agroecology of the study area was classified as arid (*Bereha*), semi-arid (*Kola*), warm sub-humid (*Woinadega*), cool-humid (*Dega*), or cold-moist (*Wurch*) based on altitude, average annual rainfall, and temperature of the study districts [21] (Table 1). The data were recorded in a Microsoft Excel spreadsheet prepared for this purpose.

**Table 1:** Traditional agroecological zones and their physical characteristics of Ethiopia

Traditional name	Agroecology	Altitude (m)	Average annual temperature (°C)	Average annual rainfall (mm)
*Bereha*	Arid	< 500	> 27.5	< 200
*Kola*	Semi-arid	500–1500/1800	20–27.5	200–800
*Woinadega*	Sub-humid	1500/1800–2300/2400	17.5/16–20	800–1200
*Dega*	Cool-humid	2300/2400–3200	11.5–17.5/16	1200–2200
*Wurch*	Cold-moist	> 3200	< 11.5	> 2200

### Data analysis

The abundance of specific tick species in infested hosts and in geographical locations was calculated by multiplying the proportion of specific ticks by 100. The number and type of studies per region and mean percent of each tick species per host, geographical location, and agroecology were presented in bar charts. Quantum GIS (QGIS) software version 3.4.5 (Open Source Geospatial Foundation, Boston, USA) was used to present the geographical distribution of individual tick species and TBPs on the map.

### Risk of bias assessment

Two volunteer individuals (see Acknowledgements section) working in different academic institutions and the author of this paper performed the literature search and data extraction from eligible studies. The literature identified was shared among individuals via email in soft copy. Data extraction was made independently by these individuals, and later validated by the author. The author of this paper crosschecked the inclusion and exclusion criteria and consistency of retrieved information to reduce the risk of bias.

## Results

### Literature search results

**Fig. 1:**
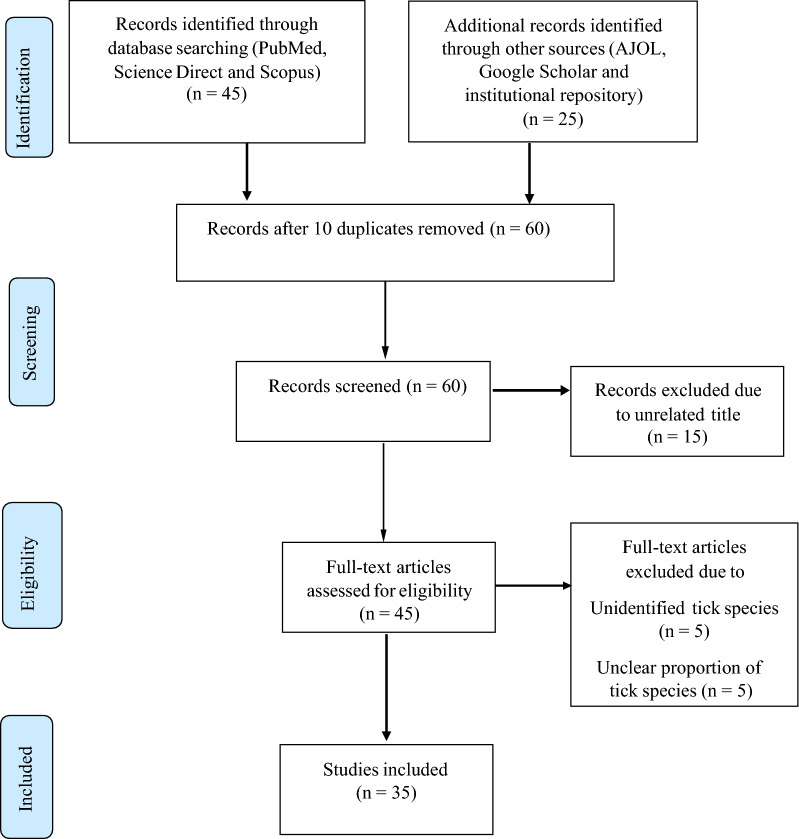
PRISMA flow diagram illustrating the process of study selection

The PRISMA flow diagram indicating the selection process for eligible studies is presented in Fig. 1. Overall, 70 studies on ixodid ticks and TBPs in domestic animals were retrieved from the electronic databases mentioned above, and were collected into Mendeley Desktop version 1.15.3. Of these, 10 duplicate studies were removed, and the screening process took place by checking the title and abstract. During the screening process, 15 studies which were unrelated to the objective of this paper were excluded, and then the rest of the studies (*n* = 45) were subjected to detailed assessment based on the eligibility criteria. Finally, 10 studies that did not meet eligibility criteria were removed, leaving only 35 studies for review.

### Characteristics of the literature

**Fig. 2:**
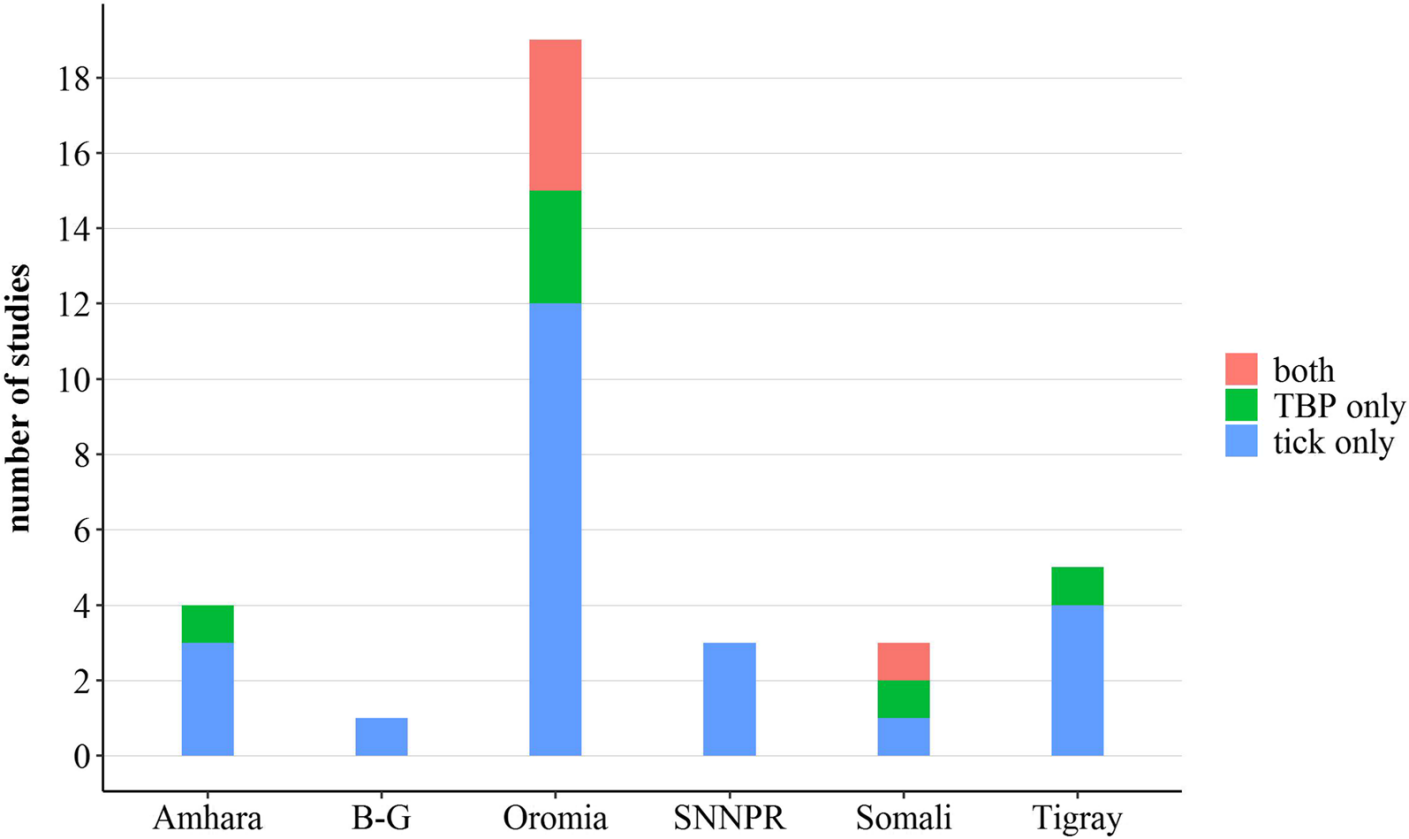
Number of study forms among administrative regions of Ethiopia

Three types of studies were identified based on the extracted data: those reporting on ixodid ticks only, TBPs only, or a combination of ticks and TBPs (both). They were from six administrative regions of the country, including Oromia (54%, *n* = 18), Amhara (11%, *n* = 4), Southern Nations, Nationalities, and Peoples’ Region (SNNPR) (9%, *n* = 3), Tigray (14%, *n* = 5), Somali (9%, *n* = 3), and Benishangul-Gumuz (B-G) (3%, *n* = 1), and one study in both Amhara and Oromia districts (3%) (Tables 2, 3). Of these, 12, three, and four studies from the Oromia region reported on ticks, TBPs, and both ticks and TBPs, respectively. In the Amhara region, three studies reported on ticks, while only one study presented data on TBPs. In the Tigray, four studies investigated ticks, while only one study reported on TBPs. No study was published regarding TBPs in the SNNPR or B-G region (Fig. 2).

**Table 2:** Literature on ixodid tick species affecting various domestic animals in different parts of Ethiopia

Administrative region	Location	Host	Tick species	References
Oromia	Central	Dogs	*Rh.* (B.) *decoloratus*, *Am. variegatum*, *Ha. leachi*, *Rh. pulchellus*, *Rh. sanguineus* s.l.	[22]
		Cats	*Am. variegatum*, *Ha. leachi*, *Rh. sanguineus* s.l.	
Oromia	Central	Horses	*Rh*. (B.) *decoloratus*, *Am. variegatum*, *Rh. pulchellus*, *Rh. evertsi evertsi*, *Am. gemma*, *Hy. rufipes*, *Hy. truncatum*	[23]
Oromia	Central	Sheep	*Rh.* (B.) *decoloratus*, *Am. variegatum*, *Rh. sanguineus* s.l., *Rh. evertsi evertsi*, *Hy. truncatum*, *Rh. pravus*, *Rh. praetextatus*	[24]
Oromia	Eastern	Cattle	*Rh*. (B.) *decoloratus*, *Am. variegatum*, *Rh. evertsi evertsi*, *Rh. pravus*, *Am. cohaerens*, *Rh. pulchellus*, *Hy. rufipes*, *Rh. bergeoni*	[25]
Oromia	Eastern	Cattle	*Rh*. (B.) *decoloratus*, *Am. variegatum*, *Rh. evertsi evertsi*, *Rh. pravus*, *Am. cohaerens*, *Rh. pulchellus*, *Hy. rufipes*, *Rh. muhsamae*, *Am. lepidum*, *Hy. truncatum*, *Rh. praetextatus*, *Am*. *gemma*	[26]
Oromia	Eastern	Camel	*Rh*. (B.) *decoloratus*, *Am. variegatum*, *Rh. pravus*, *Rh. pulchellus*, *Hy. rufipes*, *Am. lepidum*, *Am. gemma*, *Hy. dromedarii*,	[27]
Oromia	Western	Cattle	*Rh.* (B.) *decoloratus*, *Am. variegatum*, *Rh. evertsi evertsi*, *Am. cohaerens*, *Rh. pulchellus*, *Hy. rufipes*, *Rh. praetextatus*, *Am*. *gemma*	[17]
Oromia	Western	Sheep	*Rh*. (B.) *decoloratus*, *Am. variegatum*, *Rh. evertsi evertsi*, *Am. cohaerens*,	[17]
Oromia	Western	Camels	*Rh.* (B.) *decoloratus*, *Am. variegatum*, *Rh. evertsi evertsi*, *Rh. pulchellus*, *Am*. *gemma*, *Am. lepidum*, *Hy. dromedarii*, *Hy. truncatum*, *Hy. rufipes*, *Hy. excavatum*, *Hy. impeltatum*	[28]
Oromia	Western	Cattle	*Rh*. (B.) *decoloratus*, *Am. variegatum*, *Rh. evertsi evertsi*, *Am. cohaerens*, *Hy. rufipes*	[29]
Oromia	Western	Cattle	*Rh.* (B.) *decoloratus*, *Am. variegatum*, *Rh. evertsi evertsi*, *Am. cohaerens*, *Rh. lunulatus*, *Am. lepidum*	[30]
Oromia	Western	Sheep	*Rh.* (B.) *decoloratus*, *Am. variegatum*, *Rh. evertsi evertsi*, *Am. cohaerens*, *Rh. lunulatus*	[30]
Oromia	Western	Goats	*Rh*. (B.) *decoloratus*, *Am. variegatum*, *Rh. evertsi evertsi*, *Am. cohaerens*, *Rh. lunulatus*	[30]
Oromia	Western	Sheep	*Rh*. (B.) *decoloratus*, *Rh. evertsi evertsi*, *Rh. lunulatus*, *Am. lepidum*, *Am. gemma*, *Rh. pulchellus*,	[31]
Oromia	Western	Goats	*Rh.* (B.) *decoloratus*, *Rh. evertsi evertsi*, *Rh. lunulatus*, *Am. lepidum*, *Am. gemma*, *Rh. pulchellus*, *Rh. praetextatus*, *Rh. pravus*, *Am. variegatum*, *Hy. dromedarii*	[31]
Oromia	Central	Cattle	*Rh.* (B.) *decoloratus*, *Rh. evertsi evertsi*, *Am. cohaerens*, *Hy. rufipes*, *Am. variegatum*	[32]
Oromia	Eastern	Cattle	*Rh.* (B.) *decoloratus*, *Rh. evertsi evertsi*, *Am. gemma*, *Am. variegatum*, *Rh. pulchellus*	[33]
Oromia	Eastern	Sheep	*Rh.* (B.) *decoloratus*, *Rh. evertsi evertsi*, *Am. gemma*, *Am. variegatum*, *Rh. pulchellus*	[33]
Oromia	Eastern	Goats	*Rh*. (B.) *decoloratus*, *Am. gemma*, *Am. variegatum*	[33]
Oromia	Eastern	Cattle	*Rh.* (B.) *decoloratus*, *Am. gemma*, *Am. variegatum*, *Rh. pulchellus*, *Rh. evertsi evertsi*	[34]
Oromia	Eastern	Sheep	*Rh*. (B.) *decoloratus*, *Am. gemma*, *Am. variegatum*, *Rh. evertsi evertsi*	[34]
Oromia	Eastern	Goats	*Rh.* (B.) *decoloratus*, *Am. gemma*, *Am. variegatum*	[34]
Oromia	Western	Cattle	*Rh*. (B.) *decoloratus*, *Am. cohaerens*, *Am. variegatum*, *Rh. evertsi evertsi*, *Rh. praetextatus*, *Am. lepidum*, *Hy. rufipes*	[35]
Oromia	Western	Cattle	*Rh*. (B.) *decoloratus*, *Am. cohaerens*, *Am. variegatum*, *Rh. evertsi evertsi*	[12]
Oromia	Western	Cattle	*Rh.* (B.) *decoloratus*, *Am. cohaerens*, *Am. variegatum*, *Rh. evertsi evertsi*, *Rh. praetextatus*, *Am. lepidum*, *Hy. rufipes*	[36]
Amhara	Central	Horses	*Rh.* (B.) *decoloratus*, *Hy. rufipes*, *Hy. truncatum*	[23]
Amhara	Northern	Cattle	*Rh.* (B.) *decoloratus*, *Am. variegatum*, *Rh. evertsi evertsi*	[37]
Amhara	Northern	Sheep	*Rh.* (B.) *decoloratus*, *Am. variegatum*, *Rh. evertsi evertsi*, *Hy. rufipes*	[38]
Amhara	Northern	Goats	*Rh.* (B.) *decoloratus*, *Am. variegatum*, *Rh. evertsi evertsi*	[38]
Amhara	Northern	Goats	*Rh.* (B.) *decoloratus*, *Am. variegatum*, *Rh. evertsi evertsi*, *Am. gemma*	[39]
Amhara	Northern	Sheep	*Rh.* (B.) *decoloratus*, *Am. variegatum*, *Rh. evertsi evertsi*,	[39]
SNNPR	Southern	Cattle	*Rh.* (B.) *decoloratus*, *Am. variegatum*, *Rh. pulchellus*, *Rh. evertsi evertsi*, *Am. cohaerens*	[40]
SNNPR	Southern	Goats	*Rh*. (B.) *decoloratus*, *Am. variegatum*, *Rh. evertsi evertsi*	[40]
SNNPR	Southern	Donkeys	*Rh.* (B.) *decoloratus*, *Am. variegatum*	[40]
SNNPR	Southern	Horses	*Rh.* (B.) *decoloratus*, *Am. variegatum*, *Rh. pulchellus*, *Rh. evertsi evertsi*, *Rh. praetextatus*	[40]
SNNPR	Southern	Sheep	*Rh.* (B.) *decoloratus*, *Am. variegatum*, *Am. cohaerens*	[40]
SNNPR	Southern	Cattle	*Rh.* (B.) *decoloratus*, *Am. variegatum*, *Rh. evertsi evertsi*, *Am. gemma*, *Am. lepidum*, *Am. cohaerens*,	[41]
SNNPR	Southern	Sheep	*Rh.* (B.) *decoloratus*, *Am. variegatum*, *Rh. evertsi evertsi*, *Am. gemma*, *Am. lepidum*, *Am. cohaerens*	[41]
SNNPR	Southern	Goats	*Rh*. (B.) *decoloratus*, *Am. variegatum*, *Rh. evertsi evertsi*, *Am. gemma*, *Am. lepidum*, *Am. cohaerens*	[41]
SNNPR	Southern	Cattle	*Rh*. (B.) *decoloratus*, *Am. variegatum*, *Rh. evertsi evertsi*, *Am. lepidum*, *Am. cohaerens*	[42]
Tigray	Northern	Cattle	*Rh.* (B.) *decoloratus*, *Am. variegatum*, *Rh. evertsi evertsi*, *Am. gemma*, *Rh. pulchellus*, *Hy. truncatum*	[43]
Tigray	Northern	Sheep	*Rh*. (B.) *decoloratus*, *Am. variegatum*, *Rh. evertsi evertsi*, *Am. gemma*, *Rh. pulchellus*,	[44]
Tigray	Northern	Goats	*Rh.* (B.) *decoloratus*, *Am. variegatum*, *Rh. evertsi evertsi*, *Am. gemma*, *Rh. pulchellus*	[44]
Tigray	Northern	Camels	*Rh.* (B.) *decoloratus*, *Am. variegatum*, *Rh. evertsi evertsi*, *Am. gemma*, *Rh. pulchellus*, *Hy. rufipes*, *Am. lepidum*, *Am. cohaerens*, *Hy. truncatum*, *Hy. dromedarii*	[45]
Tigray	Northern	Sheep	*Rh. evertsi evertsi*, *Am. gemma*, *Am. variegatum*, *Rh.* (B.) *decoloratus*, *Hy. excavatum*	[46]
Tigray	Northern	Goats	*Rh. evertsi evertsi*, *Am. gemma*, *Am. variegatum*, *Rh*. (B.) *decoloratus*	[46]
Somali	Eastern	Cattle and camels^a^	*Rh. pravus*, *Am. gemma*, *Rh. pulchellus*, *Hy. rufipes*, *Hy. dromedarii*, *Hy. impeltatum*, *Hy. truncatum*,	[16]
Somali	Eastern	Sheep and goats^a^	*Rh. pravus*, *Rh. pulchellus*, *Hy. truncatum*	[16]
Somali	Eastern	Sheep and goats^a^	*Rh. pulchellus*, *Hy. truncatum*, *Am. variegatum*, *Rh. evertsi evertsi*, *Am. gemma*, *Rh. pulchellus*, *Hy. rufipes*	[47]
B-G	Western	Cattle	*Rh. pulchellus*, *Am. variegatum*, *Rh. evertsi evertsi*, *Hy. rufipes*, *Am. lepidum*, *Rh.* (B.) *decoloratus*	[48]

^a^Hosts affected by all tick species mentioned under row

**Table 3:** Reports on tick-borne pathogens in animal or tick species collected from hosts in different areas of Ethiopia

Administrative region	Location	Animal/tick	Host from which ticks were collected	TBPs	Technique of detection	References
Oromia	Western	*Rh. evertsi evertsi*, *Rh.* (B.) *decoloratus*	Sheep and cattle	*A. ovis* *Ehrlichia* sp., *Anaplasma* sp. *Rickettsia* sp. *E. ruminantium* *R. africae*	Molecular	[49]
Oromia	Western	Cattle	–	*A. centrale* *A. marginale* *B. bigemina*	Thin blood smear	[12]
Somali	Eastern	*Am. gemma* *Hy. impeltatum* *Hy. truncatum* *Hy. rufipes*	Cattle, sheep, and goats	*R. africae* *R. aeschlimannii*	Molecular	[50]
Somali	Eastern	*–*	Cattle, camels	*T. velifera* *T. mutans*	Molecular	[16]
Somali	Eastern	*Am. gemma*	Cattle	*E. ruminantium*	Molecular	[16]
Amhara	Northern	Cattle	*–*	*T. orientalis*	Molecular	[51]
Amhara	Northern	Cattle and sheep	*–*	*T. velifera* *T. mutans* *T. ovis*	Molecular	[52]
Tigray	Northern	Cattle, goats, and sheep	*–*	*T. velifera* *T. mutans* *T. annulata* *T. ovis* *T. separate*	Molecular	[52]
Oromia	Western	*Rh.* (B.) *decoloratus* *Rh. evertsi evertsi*	Cattle and sheep	*T. ovis* *Theileria* sp.	Molecular	[17]
Oromia	Western	*Hy. rufipes*	Cattle	*Francisella*-like endosymbiont	Molecular	[53]
Oromia	Western	*Am. variegatum* *Am. cohaerens*	Cattle	*E. ruminantium*, *A. phagocytophilum*, *A. marginale*	Molecular	[35]
Oromia	Western	*Am. variegatum* *Am. cohaerens*	Cattle	*C. burnetii*	Molecular	[36]
Oromia	Western	Cattle and sheep	*–*	*A. phagocytophilum*	Molecular	[15]
Oromia	Southern	Cattle and goats	*–*	*A. marginale* *A. centrale* *Anaplasma* sp. ‘Omatjenne’	Molecular	[15]
Oromia	Central	Cattle, sheep, and goats	*–*	*A. marginale* *Anaplasma* sp. ‘Omatjenne’ *A. phagocytophilum* *A. centrale* *A. ovis*	Molecular	[15]

Ticks were collected from different domestic animals, including cattle, donkeys, horses, sheep, goats, camels, dogs, and cats. TBPs were detected from either the blood of the host or tick tissue. Molecular techniques (conventional polymerase chain reaction [PCR] and gene sequencing) were employed for the identification of TBPs in all reports but one [12]. No study was documented regarding TBPs in SNNPR, despite the fact that the region is endemic to various ixodid ticks. As regards geographical location, ticks and TBPs appear to occur in almost all locations, including central, southern, eastern, western, and northern Ethiopia, although certain variations in terms of species-specific burden are expected to exist. Indeed, many biotope factors can influence the burden of specific tick species. Hence, a difference was observed in the abundance of tick species among four traditional agroecological zones (arid, semi-arid, warm sub-humid, and cool-humid) of the country.

### Distribution of ixodid tick species

#### *Amblyomma variegatum*

**Fig. 3:**
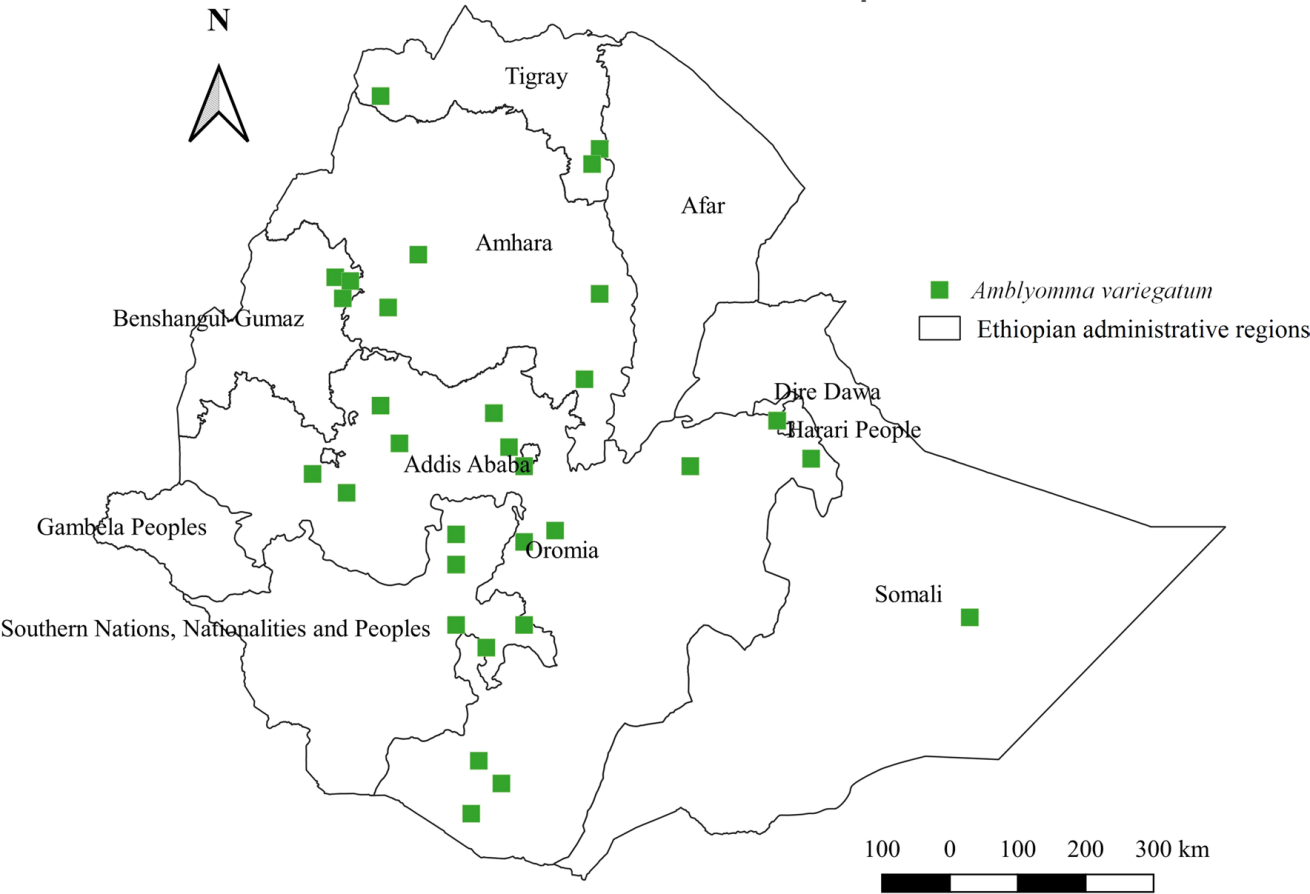
Distribution of *Am. variegatum* in Ethiopia

*Amblyomma variegatum* infests a wide range of domestic animals including cattle, donkeys, horses, camels, dogs, cats, sheep, and goats. However, the infestation burden was highest in donkeys, followed by cattle, goats, sheep, dogs, horses, camels, and cats (Additional file 1: Fig. S1). Regarding distribution, it is highly abundant in the western region of the country, followed by central, southern, eastern, and northern Ethiopia, characterized by either warm sub-humid or cool-humid agroecology (Additional file 2: Fig. S2, Additional file 3: Fig. S3). It has been reported from all administrative regions of the country except Gambela and Afar (Fig. 3).

#### *Amblyomma gemma*

**Fig. 4:**
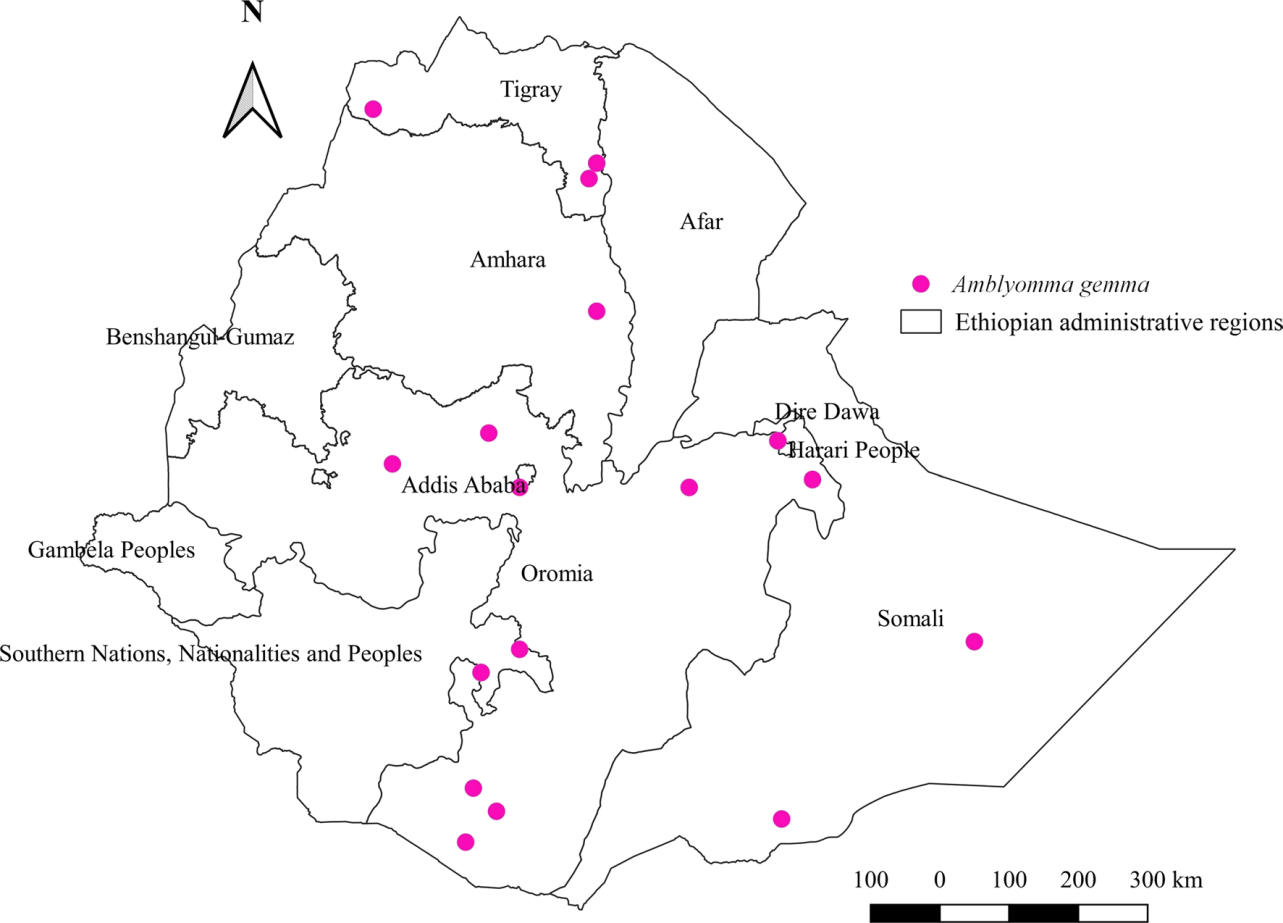
Distribution of *Am. gemma* in Ethiopia

According to this review, *Am. gemma* affects cattle, horses, sheep, goats, and camels. However, the highest burden of this tick was collected from goats, followed by sheep, cattle, camels, and horses (Additional file 1: Fig. S1). Concerning distribution, the abundance of *Am. gemma* relative to other species seems to be highest in the eastern region of the country, followed by southern, northern, central, and western regions (Additional file 2: Fig. S2). The highest percent of this tick was observed in arid or semi-arid agroecology (Additional file 3: Fig. S3). It was identified in five regional states of Ethiopia, namely Amhara, Tigray, Oromia, SNNPR, and Somali (Fig. 4).

#### *Amblyomma lepidum*

**Fig. 5:**
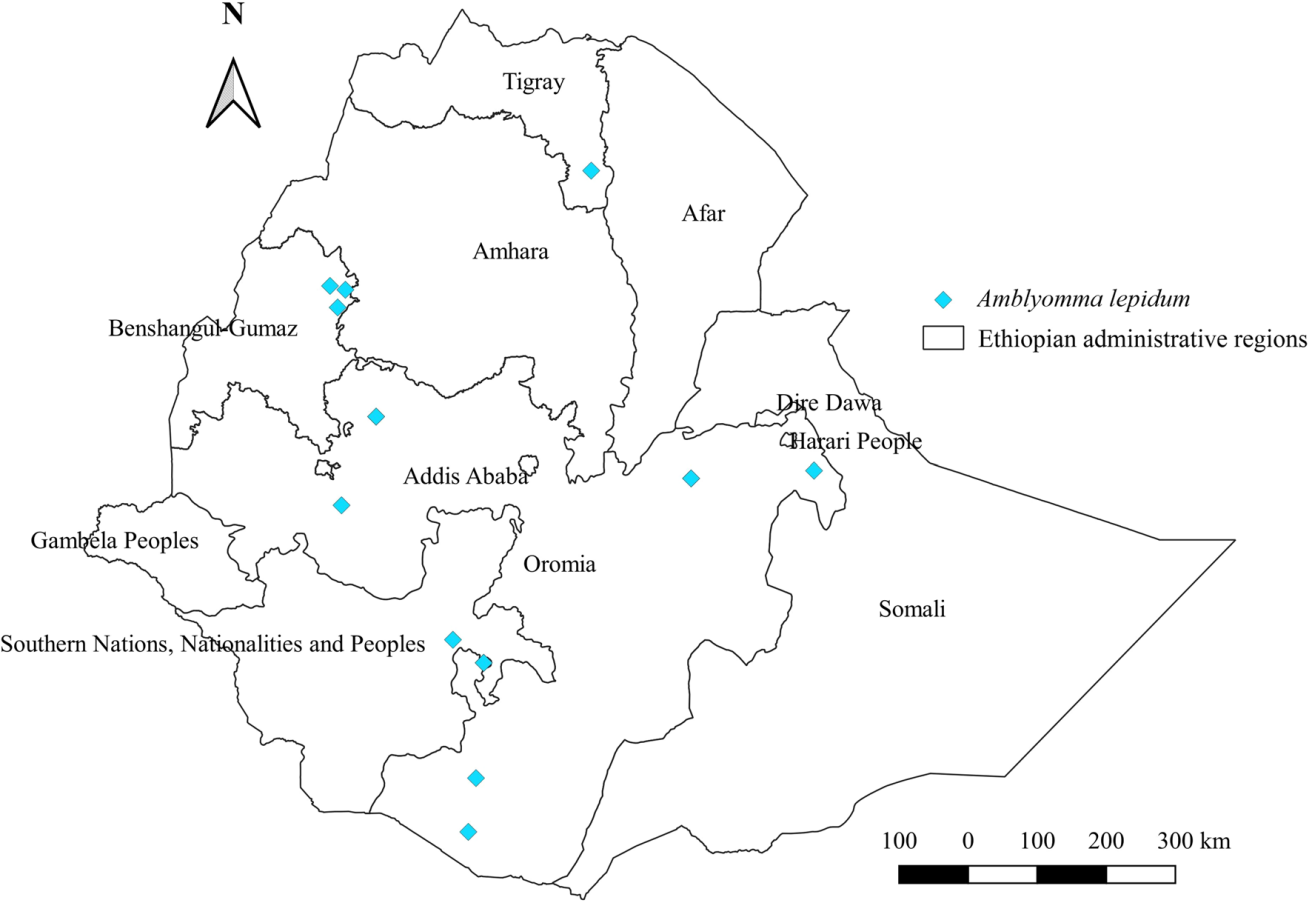
Distribution of *Am. lepidum* in Ethiopia

*Amblyomma lepidum* has been found to affect four types of domestic animals in Ethiopia. These animals are cattle, sheep, goats, and camels, of which goats appear to be the most preferred host, followed by sheep, cattle, and camels (Additional file 1: Fig. S1). Its distribution is restricted to eastern, southern, and western geographical locations. Nevertheless, its relative abundance seems highest in the eastern region of the country, which is characterized by semi-arid agroecology (Additional file 2: Fig. S2, Additional file 3: Fig. S3). This tick has been reported from Oromia, SNNPR, Tigray, and B-G regional states of the country (Fig. 5).

#### *Amblyomma cohaerens*

**Fig. 6:**
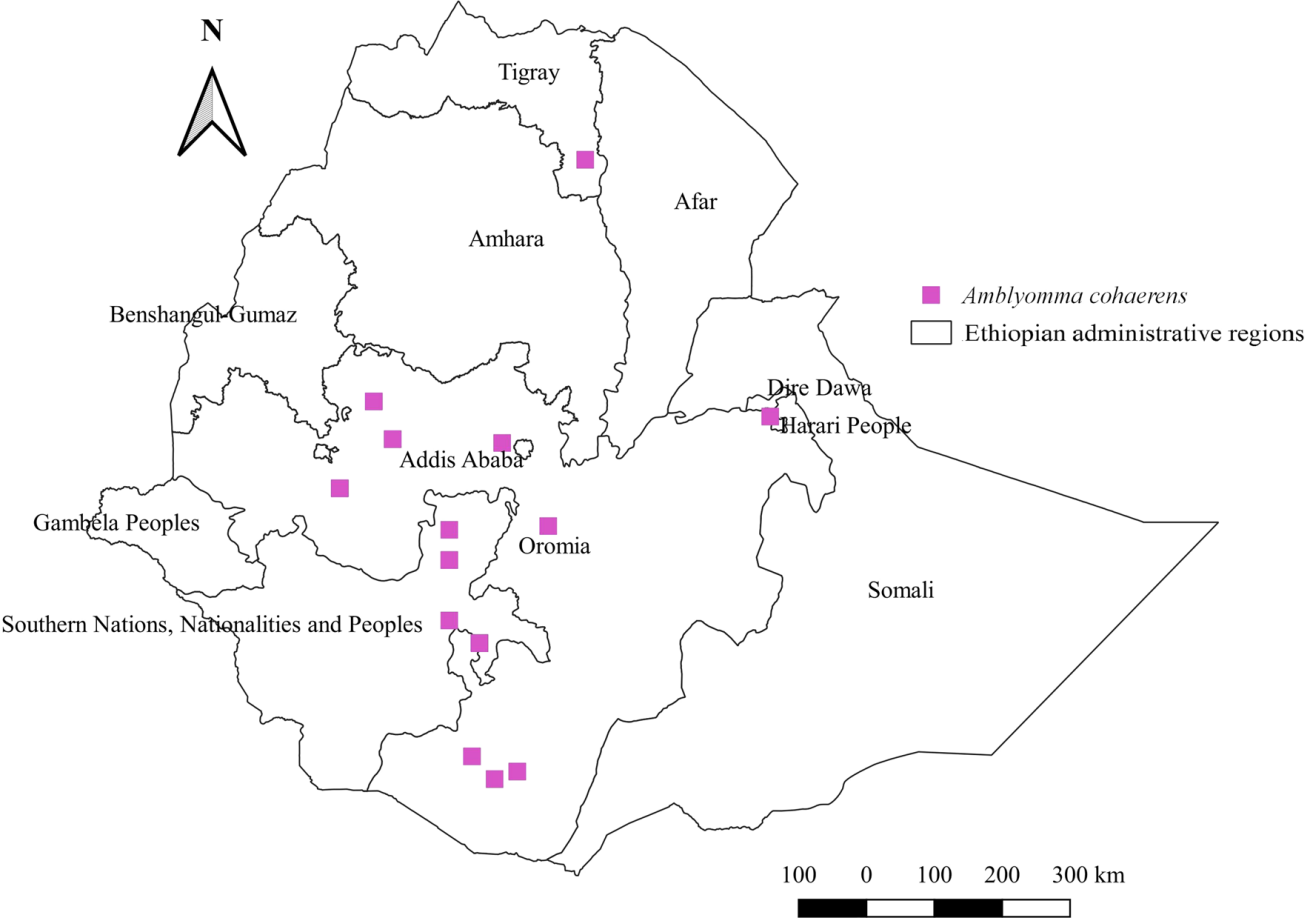
Distribution of *Am. cohaerens* in Ethiopia

*Amblyomma cohaerens* affects cattle, sheep, goats, and camels. The highest abundance of this tick was observed in cattle, followed by sheep, goats, and camels (Additional file 1: Fig. S1). This tick has been reported from only three administrative regions, namely Oromia, Tigray, and SNNPR. It is the most abundant and widely distributed tick in western Oromia (Additional file 2: Fig. S2). The tick performs best in semi-arid and sub-humid agroecologies (Additional file 3: Fig. S3). Its distribution covers central and southern Oromia, southern parts of SNNPR, eastern and western Oromia, and northern locations (Fig. 6).

#### *Hyalomma truncatum*

**Fig. 7:**
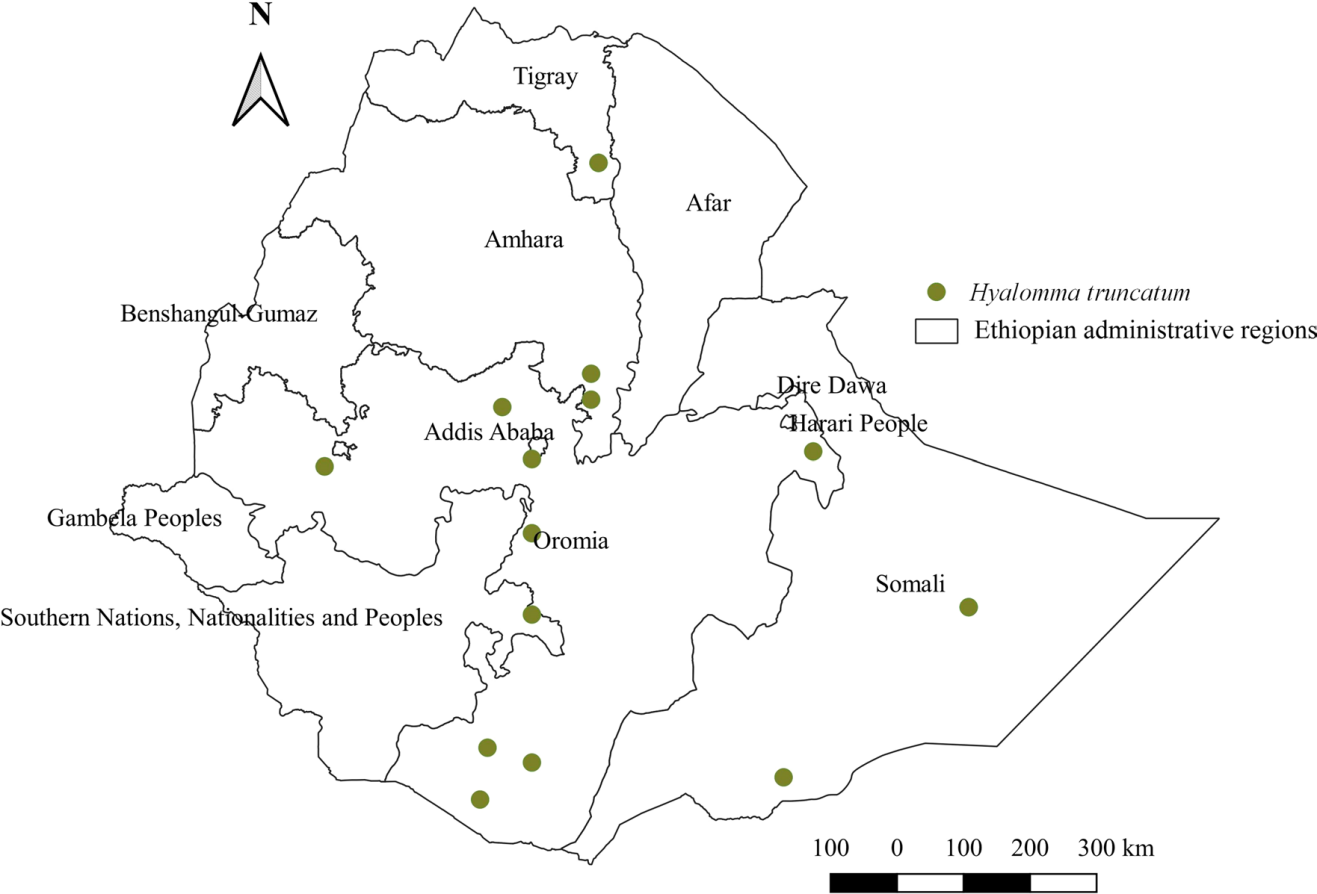
Distribution of *Hy. truncatum* in Ethiopia

*Hyalomma truncatum* has been identified from cattle, horses, sheep, goats, and camels. However, the highest abundance was identified in goats (Additional file 1: Fig. S1). This tick circulates in almost all geographical locations of various agroecological zones, although its abundance seems to be greatest in arid lowlands of eastern locations (Additional file 2: Fig. S2 and Additional file 3: Fig. S3). It also occupies southern parts of Tigray regional states, central, western, and southern Oromia, and SNNPR states (Fig. 7).

#### *Hyalomma rufipes*

**Fig. 8:**
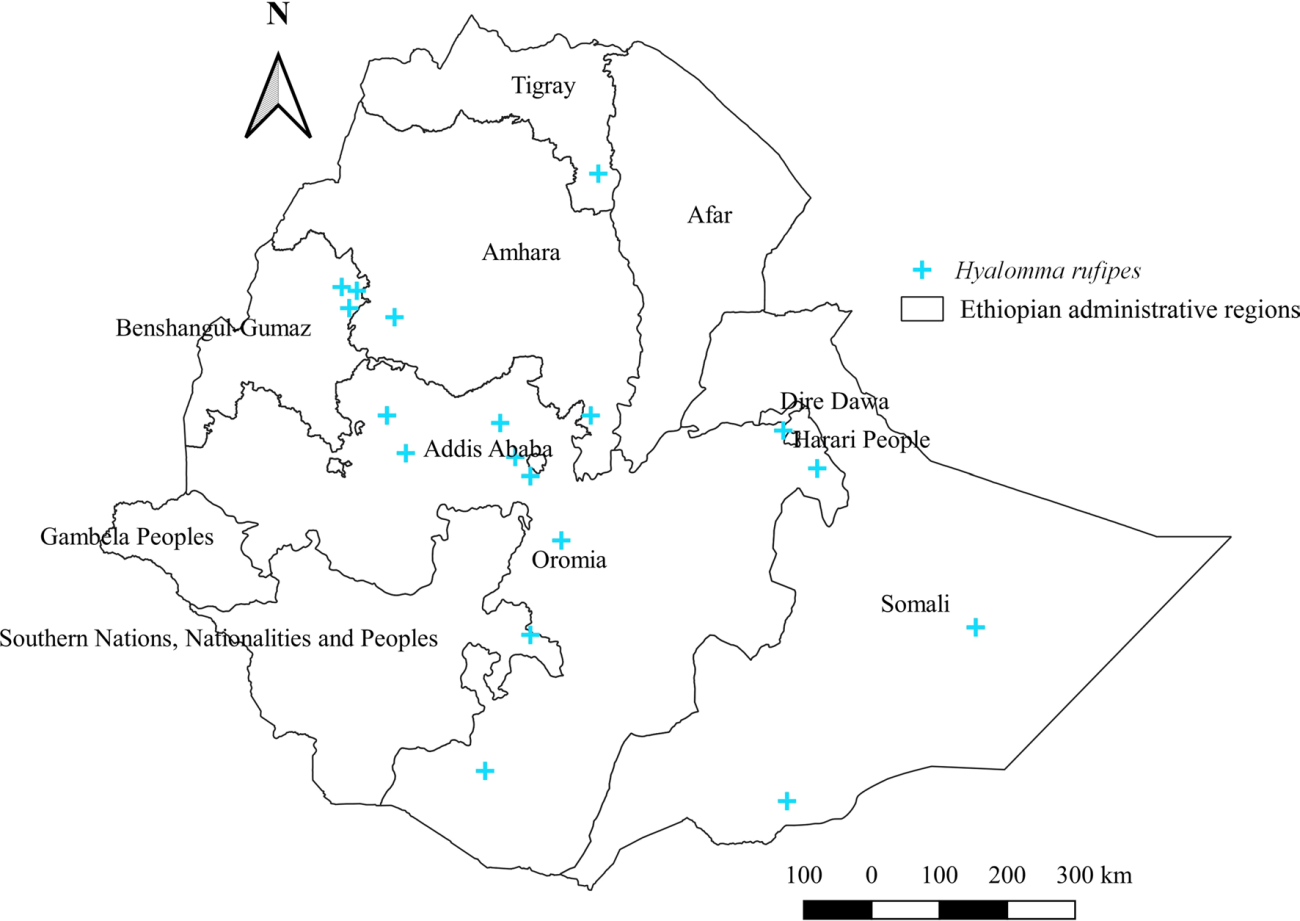
Distribution of *Hy. rufipes* in Ethiopia

*Hyalomma rufipes* originally belonged to the subgenus *marginatum* and hence was named *Hy. marginatum rufipes*. Scientists eventually discriminated *Hy. rufipes* from *Hy. marginatum*, and now these ticks stand as an independent tick species. *Hyalomma rufipes* has been collected and identified from cattle, horses, sheep, goats, and camels in Ethiopia. Of these hosts, goats are the most commonly affected by *Hy. rufipes* (Additional file 1: Fig. S1). It is the most widely distributed species of the genus *Hyalomma* in the country and occupies central, northwestern, southern, and eastern locations (Fig. 8). Although reports concerning this tick are few in the eastern region, its relative abundance is high in this area (Additional file 2: Fig. S2). And while it thrives in any agroecological zone, it performs best in arid agroecology (Additional file 3: Fig. S3).

#### *Hyalomma dromedarii*

**Fig. 9:**
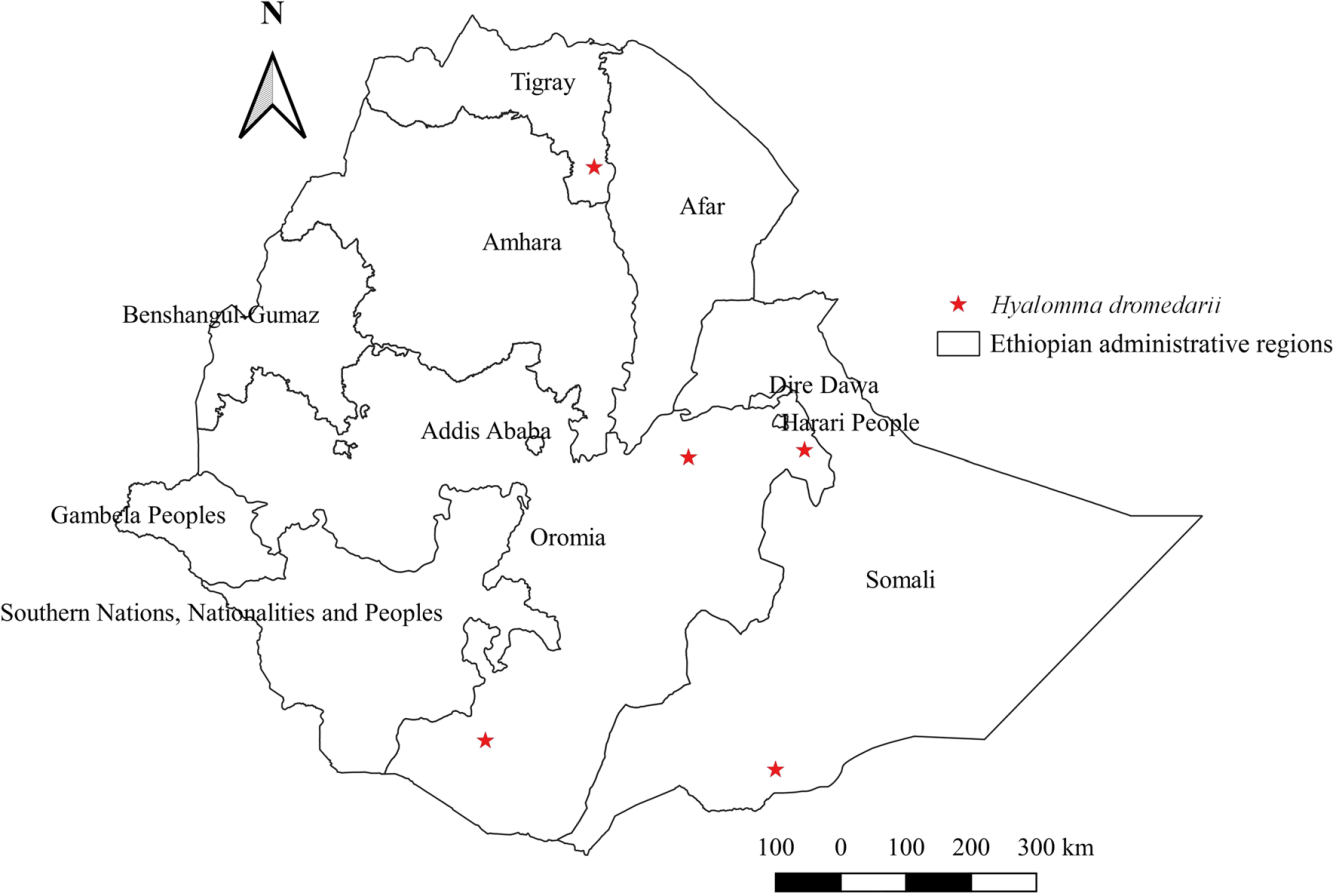
Distribution of *Hy. dromedarii* in Ethiopia

*Hyalomma dromedarii* affects a wide range of domestic animals, including camels, cattle, and goats. However, the highest relative abundance was observed in camels (Additional file 1: Fig. S1). In Ethiopia, this tick was reported from the eastern area of Somali and Oromia, southern Oromia, and southern parts including Tigray. Nonetheless, the highest abundance was demonstrated in northern and eastern areas that have semi-arid agroecological features (Additional file 2: Fig. S2, Additional file 3: Fig. S3; Fig. 9).

#### *Rhipicephalus pulchellus*

**Fig. 10:**
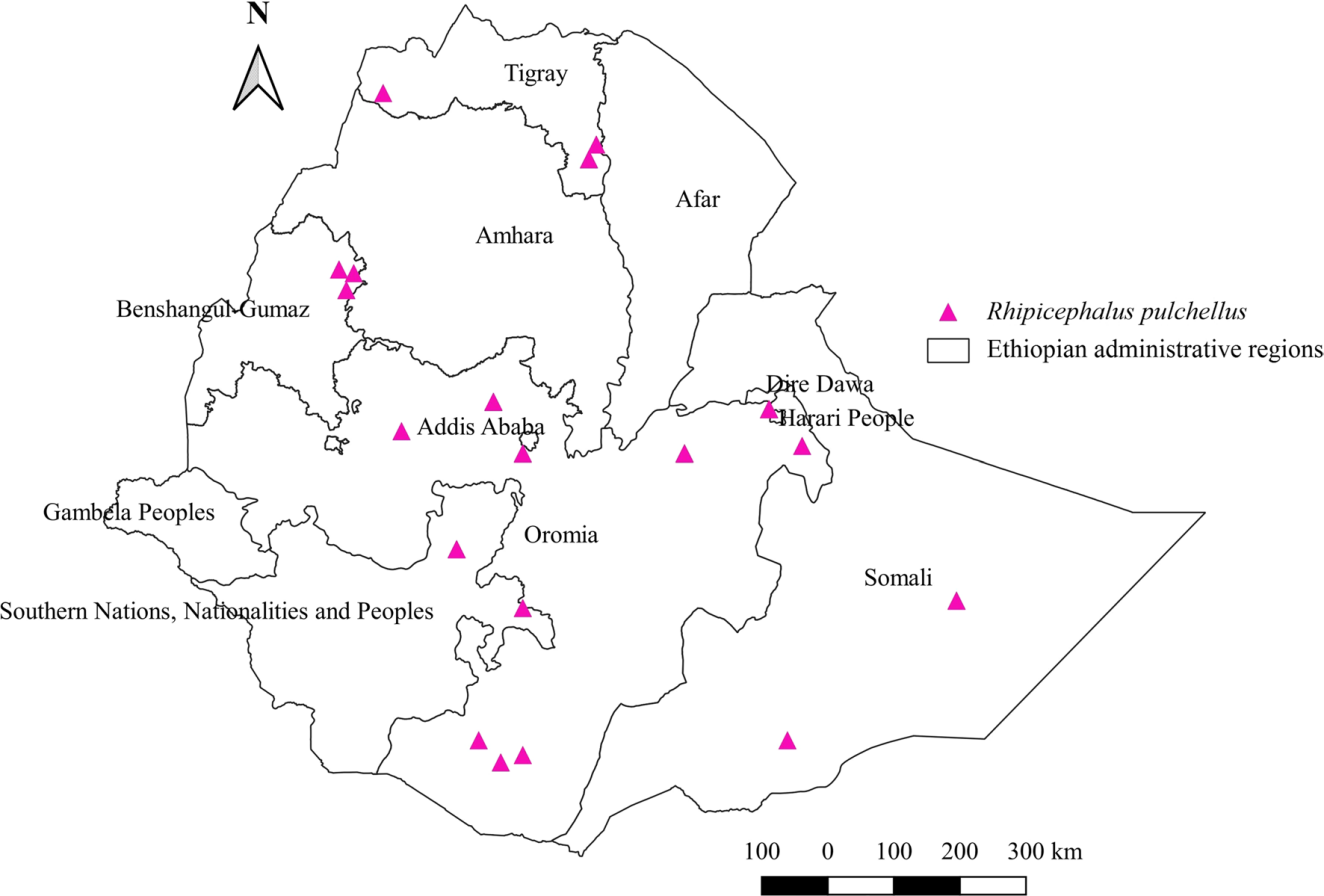
Distribution of *Rh. pulchellus* in Ethiopia

*Rhipicephalus pulchellus* was identified in all domestic animals except cats. The highest relative percent was observed in camels, followed by cattle, goats, sheep, horses, donkeys, and dogs (Additional file 1: Fig. S1). It covers a wide range of geographical areas, including central, southern, eastern, western, and southern locations. The highest relative abundance of this tick was seen in the north, followed by the southern, eastern, central, and western parts of the country (Additional file 2: Fig. S2). Its occurrence was confirmed in all administrative regions with semi-arid lowland agroecology except Gambela and Afar (Additional file 3: Fig. S3, Fig. 10).

#### *Rhipicephalus evertsi evertsi*

**Fig. 11:**
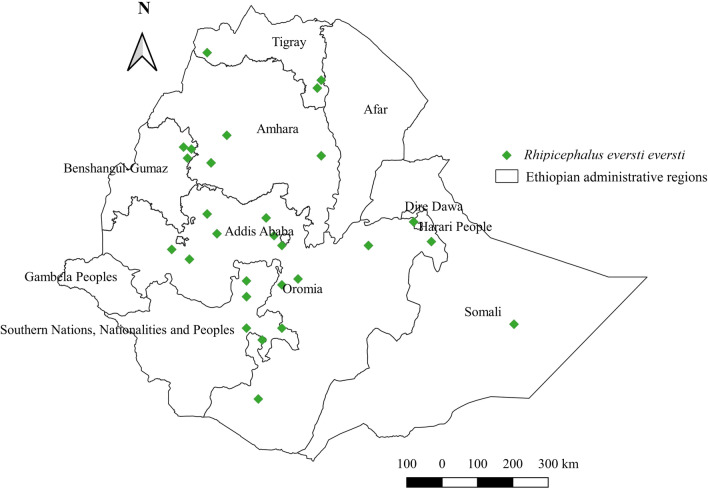
Distribution of *Rh. evertsi evertsi* in Ethiopia

*Rhipicephalus evertsi evertsi* has been found to infest cattle, horses, donkeys, sheep, goats, and camels in Ethiopia. The highest relative number was identified in horses, followed by sheep, goats, donkeys, cattle, and camels (Additional file 1: Fig. S1). This tick is reported from all administrative regions except Gambela and Afar, and from all geographical locations, including central, southern, eastern, northern, and western areas (Fig. 11). Its relative abundance is highest in western regions of Ethiopia, which are characterized by a warm sub-humid and cool-humid agroecology (Additional file 2: Fig. S2, Additional file 3: Fig. S3).

### *Rhipicephalus* (Boophilus) *decoloratus*

**Fig. 12:**
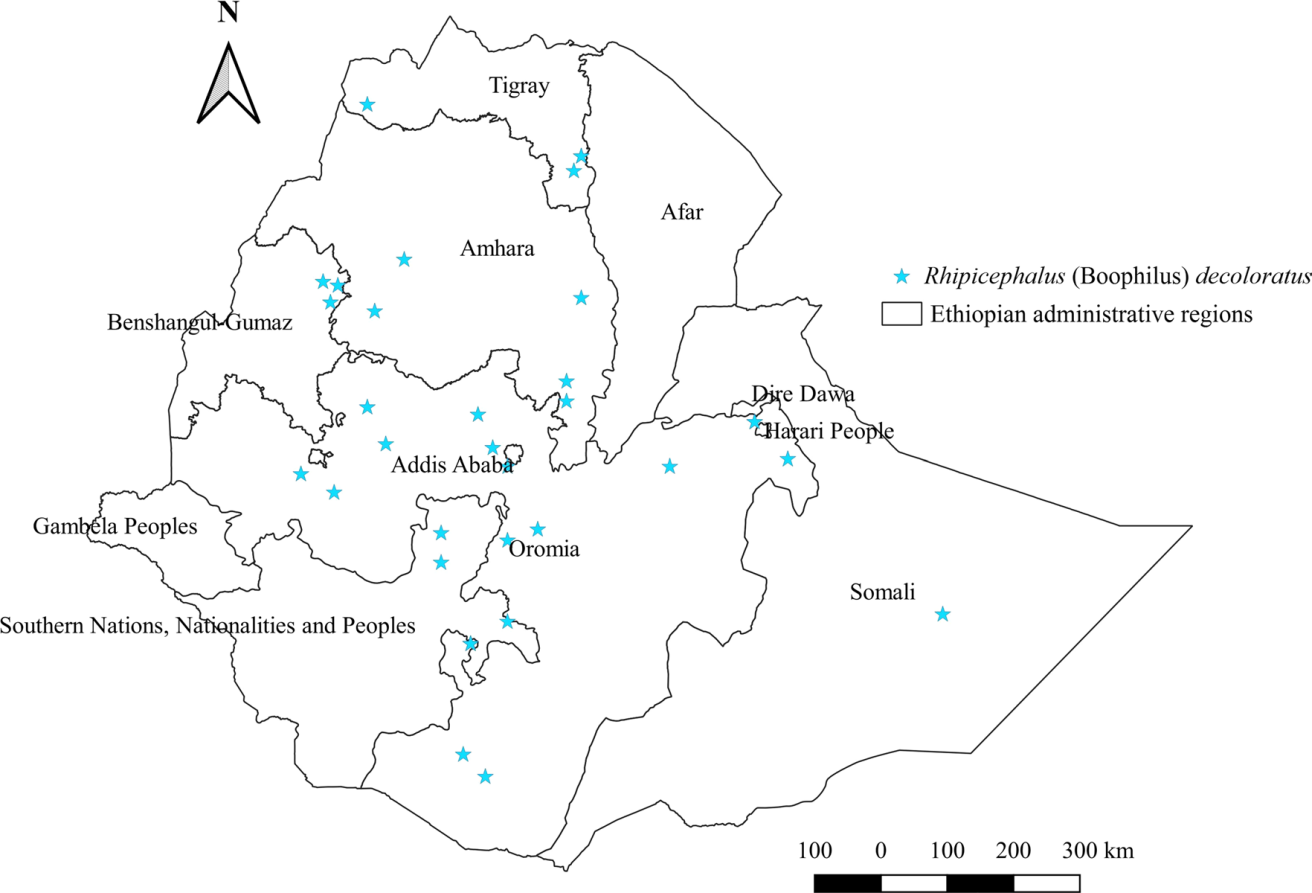
Distribution of *Rh.* (B.) *decoloratus* in Ethiopia

*Rhipicephalus* (Boophilus) *decoloratus* affects all domestic animals included in this report except cats. Donkeys are the host most preferred by this tick, followed by goats, cattle, sheep, horses, camels, and dogs (Additional file 1: Fig. S1). It occupies wide geographical locations, including central, southern, eastern, northern, and western parts of the country (Fig. 12). However, the highest abundance was estimated in southern areas, characteristically being a warm sub-humid area (Additional file 2: Fig. S2, Additional file 3: Fig. S3).

### *Rhipicephalus sanguineus* sensu lato

**Fig. 13:**
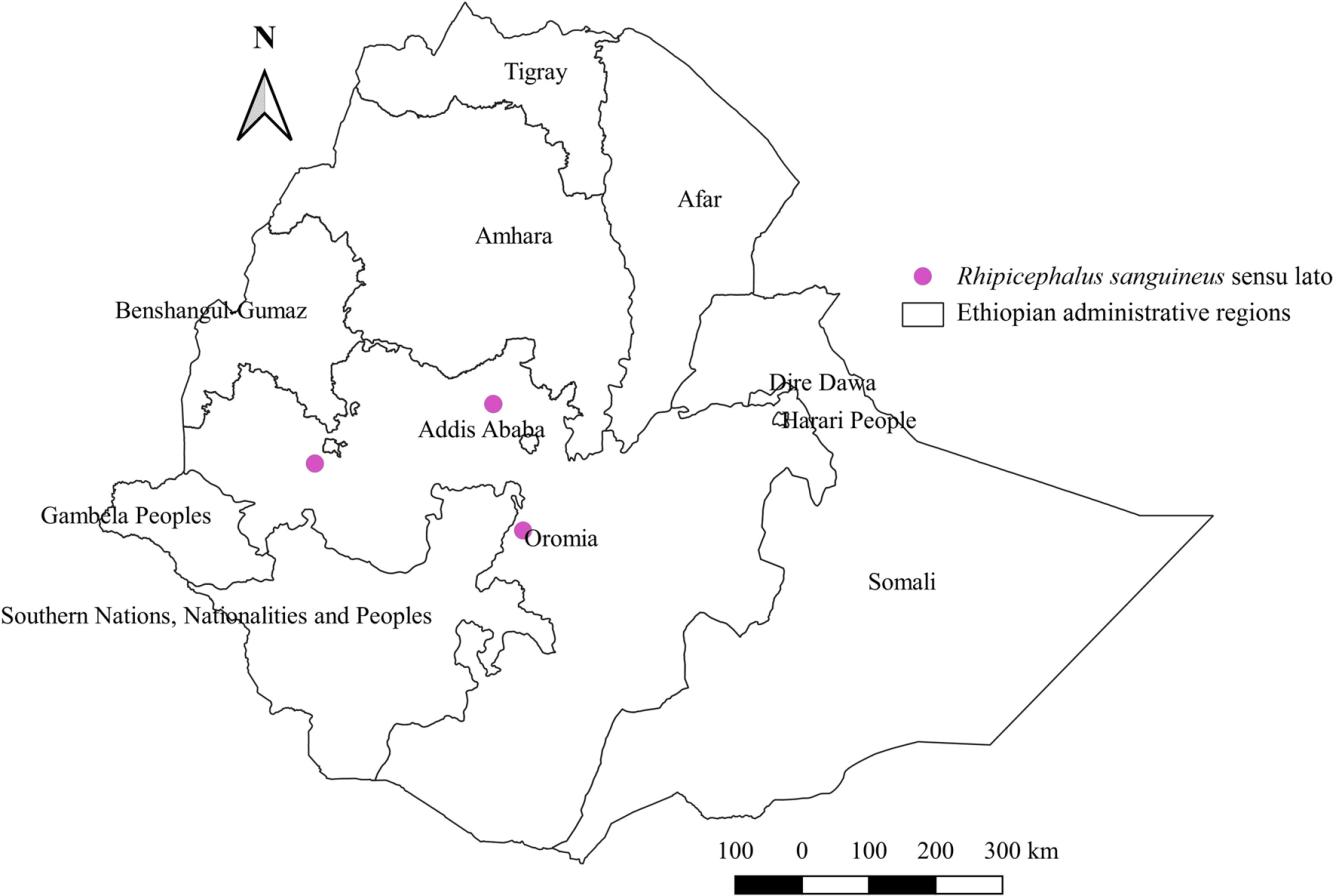
Distribution of *Rh. sanguineus* s.l. in Ethiopia

*Rhipicephalus sanguineus* s.l. is a common tick that infests domestic dogs worldwide. Our review findings reveal that this tick not only affects dogs, but also affects donkeys, sheep, and cats. As was expected, dogs were found to harbor the highest relative burden (Additional file 1: Fig. S1). It was reported only from the central areas of the country with a warm sub-humid agroecology (Additional file 2: Fig. S2, Additional file 3: Fig. S3, Fig. 13).

#### *Rhipicephalus pravus*

**Fig. 14:**
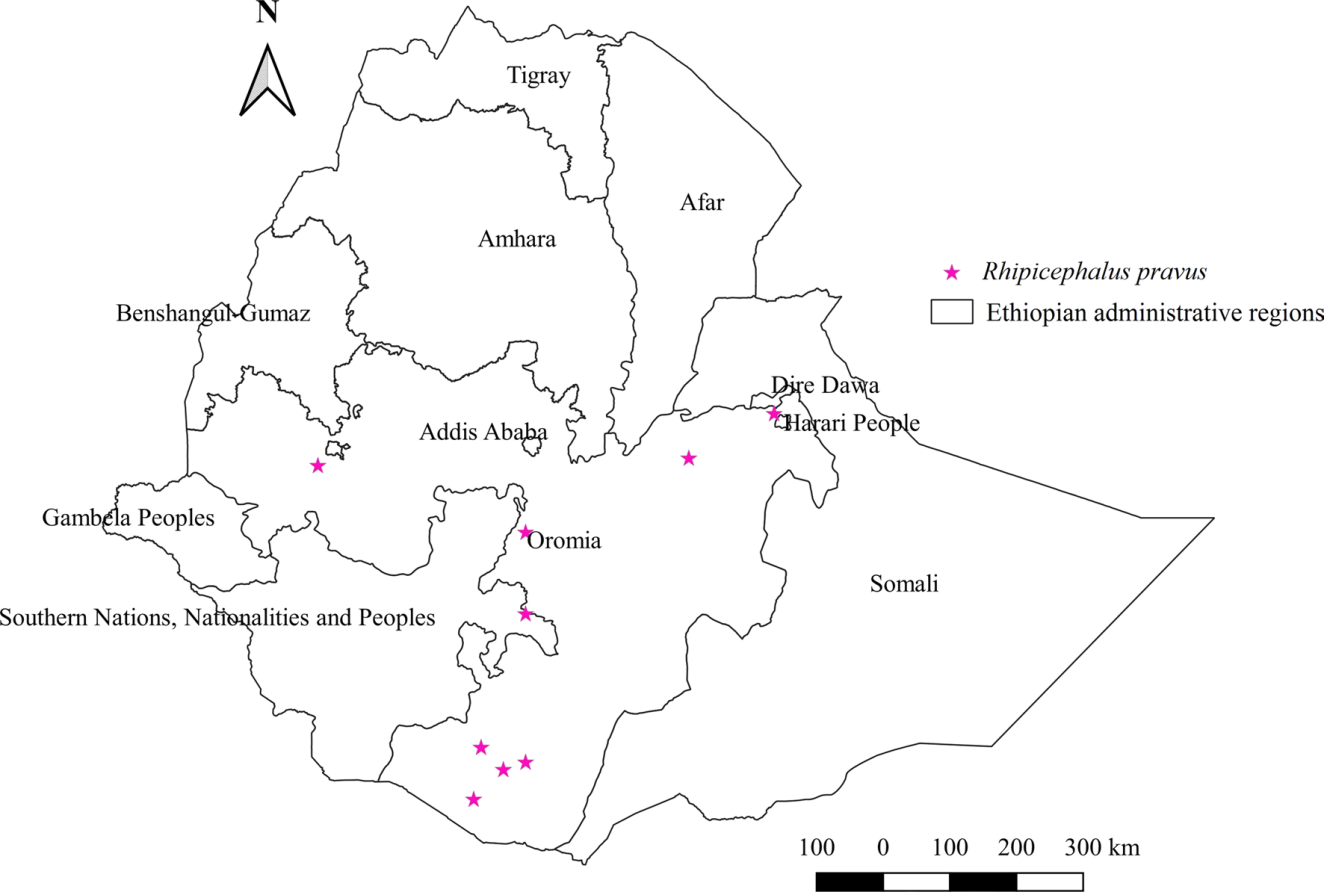
Distribution of *Rh. pravus* in Ethiopia

In Ethiopia, *Rh. pravus* was collected from cattle, sheep, and goats; however, cattle were the most frequently infested animals (Additional file 1: Fig. S1). It was reported from central, southern, and eastern geographical locations, and its relative abundance was highest in the eastern region (Additional file 2: Fig. S2, Fig. 14). The arid lowland is a suitable agroecology for the biology of this tick (Additional file 3: Fig. S3).

#### *Rhipicephalus lunulatus*

**Fig. 15:**
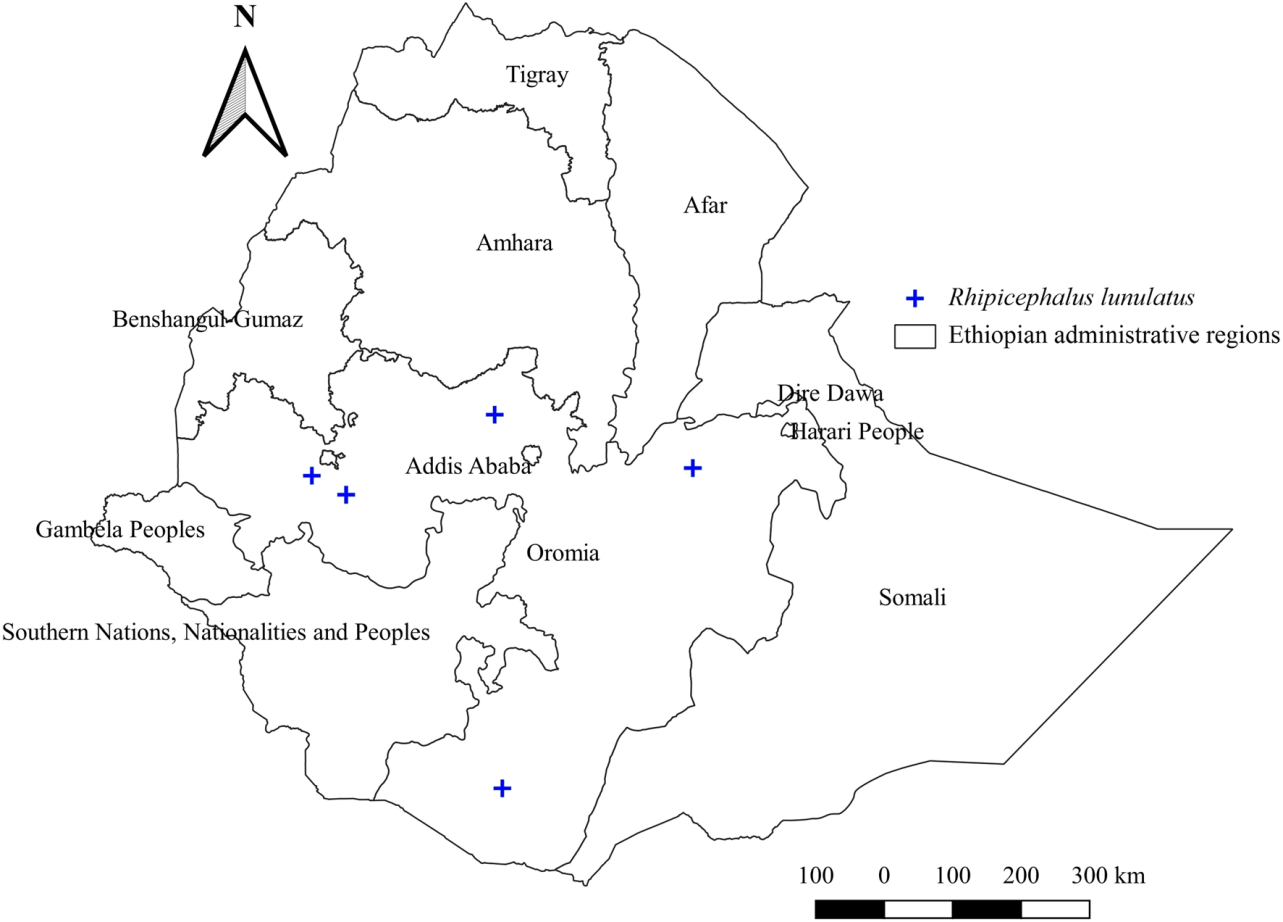
Distribution of *Rh. lunulatus* in Ethiopia

*Rhipicephalus lunulatus* was collected from cattle, sheep, goats, and donkeys. It was reported from the central, southern, eastern, and western parts of Oromia (Fig. 15). Central and eastern regions, which have a warm sub-humid agroecology, appear to have a higher relative abundance of this tick (Additional file 2: Fig. S2, Additional file 3: Fig. S3).

#### *Rhipicephalus muhsamae*

**Fig. 16:**
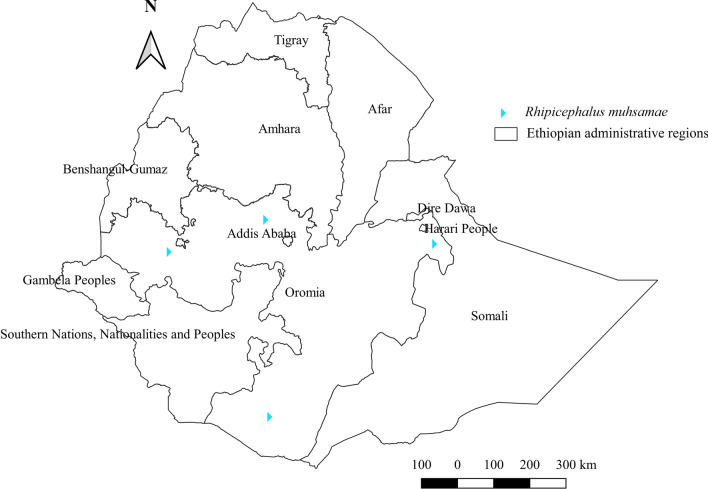
Distribution of *Rh. muhsamae* in Ethiopia

*Rhipicephalus muhsamae* was identified in cattle and donkeys. Higher relative infestation by this tick was observed in donkeys (Additional file 1: Fig. S1). It was reported from the semi-arid climate of the central, southern, and eastern Oromia regions (Additional file 2: Fig. S2, Additional file 3: Fig. S3, Fig. 16).

#### *Rhipicephalus praetextatus*

**Fig. 17:**
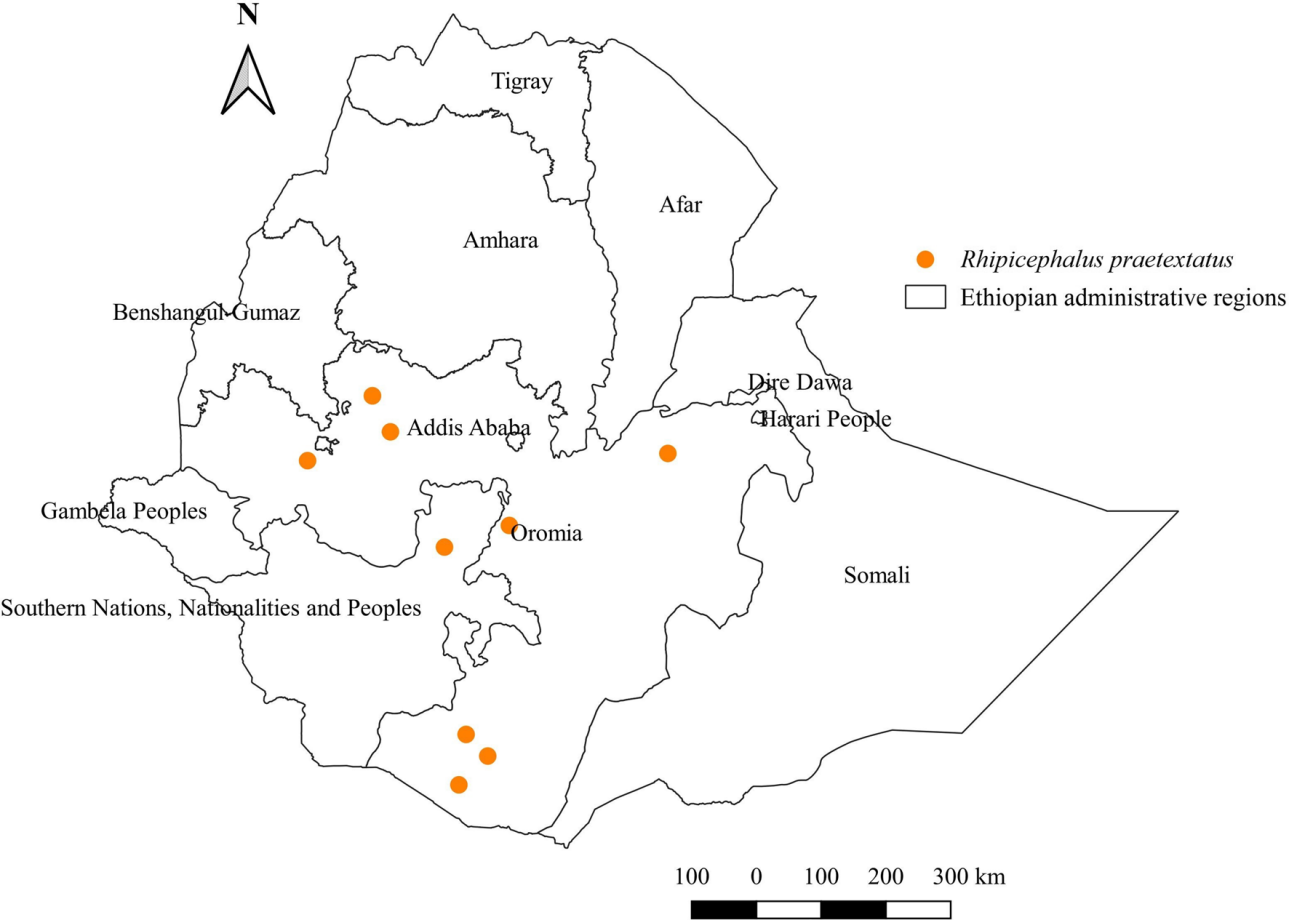
Distribution of *Rh. praetextatus* in Ethiopia

*Rhipicephalus praetextatus* was detected in cattle, sheep, goats, and horses. The highest proportion was observed in horses (Additional file 1: Fig. S1). It was reported from central, southern, eastern, and western locations of two administrative regions, Oromia and SNNPR (Fig. 17). However, the highest abundance was observed in the eastern geographical locations characterized by semi-arid and sub-humid agroecologies (Additional file 2: Fig. S2, Additional file 3: Fig. S3).

#### *Hyalomma excavatum*, *Hyalomma impeltatum*, *Haemaphysalis leachi*, and *Rhipicephalus bergeoni*

**Fig. 18:**
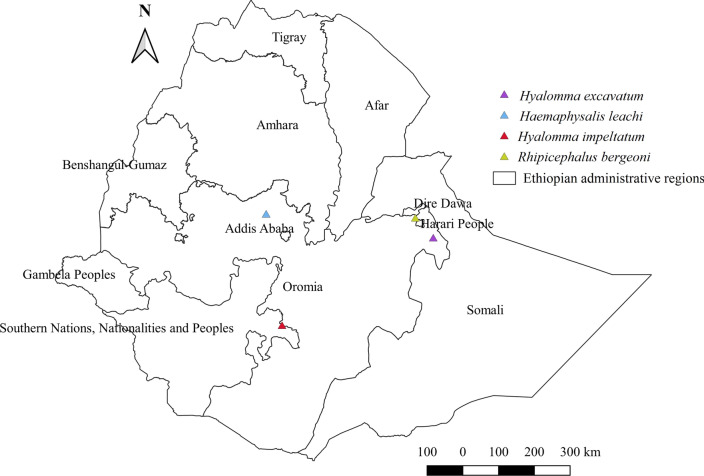
Distribution of *Hy. excavatum*, *Hy. impeltatum*, *Ha. leachi*, and *Rh. bergeoni* in Ethiopia

Certain less frequently encountered species of the genera *Hyalomma*, *Rhipicephalus*, and *Haemaphysalis* have been seldom reported in the country (Fig. 18). *Hyalomma excavatum* was reported from cattle in the eastern Oromia region. *Hyalomma impeltatum* was reported on cattle and camels in the SNNPR region and eastern part of Oromia. The historical report of *Rhipicephalus bergeoni*, an unfamiliar tick, in the central and eastern parts of the country [19, 28], while this tick is largely missing in other locations, remains an area of interest. *Haemaphysalis leachi* is a relatively better-reported tick of the genus *Haemaphysalis* when compared with other species. It has been found to infest dogs and cats in central Ethiopia [22].

### Distribution of tick-borne pathogens (TBPs)

**Fig. 19:**
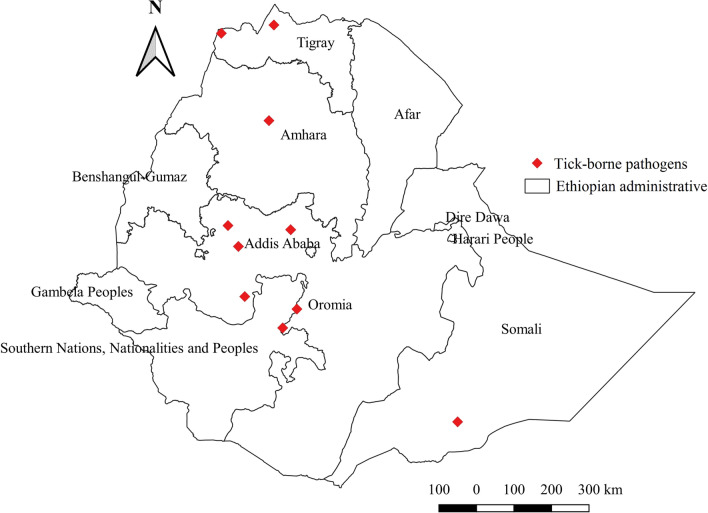
Distribution of tick-borne pathogens in Ethiopia

Reports on ixodid ticks are on the rise, but studies on tick vector competence and associated pathogens that they transmit to animals are scant in the country. Of 11 administrative regions, TBPs have been reported from only four regions, namely Oromia, Amhara, Somali, and Tigray. TBPs were identified from either tick tissue or animal biological samples using molecular techniques, and several cases of *Anaplasma*, *Ehrlichia*, *Rickettsia*, and *Theileria* species, *Babesia bigemina*, *Francisella*-like endosymbiont, and *Coxiella burnetii* were diagnosed (Table 3). Almost all studies on TBPs are from the western region, with few reports from the central, eastern, and southern parts of Ethiopia (Fig. 19).

## Discussion

This paper was designed to summarize and compile available data on the distribution and relative abundance of ixodid tick species in domestic animals in Ethiopia. It also aimed to present information on the TBPs and their distribution in the country. Accordingly, the author reports the presence of several ixodid ticks in almost all administrative regions of the country owing to the suitability of Ethiopian agroecologies to their survival and transmission. Despite this, an extensive report from Oromia shows that the region has the largest land cover with suitable agroecology for agricultural practices, including livestock production. Meanwhile, in the administrative regions of Gambela and Afar, reports on ixodid tick or TBPs are missing. Tick surveillance in these regions, particularly in Gambela, is of paramount importance, as this region borders South Sudan, where *Rh. appendiculatus*, the vector of *Theileria parva*, is prevalent [55].

### Ixodid ticks: distribution, abundance, and affected hosts

#### *Amblyomma variegatum*

The occurrence of *Am. variegatum* in all geographical locations of the country suggests that many agroecological zones in the country favor its breeding. The current report showing *Am. variegatum* to be the most abundant tick in western regions of Ethiopia indicates that the warm sub-humid agroecology of the region favors its biology. This finding is in accord with a report from Cameroon [56] demonstrating the occurrence of this tick on cattle in all surveyed sites and its highest abundance in warm savanna degraded forest. On the contrary, a study conducted in western Kenya [57] reported the absence of *Am. variegatum* on cattle and buffalo in the surveyed region. This could be due to the unfavorable agroecological zone of the study regions, pointing to the impacts of climate variability on tick distribution. Regarding the affected host, it appears that no domestic animal is spared from infestation by *Am. variegatum*. Although this report does not present the infestation status of wildlife, a report of this tick on wild rhinoceroses in Zambia [58] reveals its non-host-specific feeding characteristic. However, its highest burden in donkeys in the current report warrants high control practices against this tick and associated pathogens in the region’s donkey population.

#### *Amblyomma gemma*

The highest abundance of *Am. gemma* in eastern arid and semi-arid agroecologies reveals its drought-resistant trait, which allows the tick to prevail throughout the year in the region. The highest burden of this tick in goats according to the current review points to the usual production system of goats in areas of the country that are characteristically arid or semi-arid, where this tick exists. This finding is in line with a study conducted in a pastoral community of Kenya [59] that shows the infestation of ruminants and camels by *Am. gemma*. This tick is non-host-specific, as it can feed on any animals, including wildlife.

#### *Amblyomma lepidum*

*Amblyomma lepidum* usually coexists with other *Amblyomma* species in the country, particularly *Am. gemma*. This is evidenced by its highest abundance in arid and semi-arid agroecologies of eastern locations coupled with its highest infestation rate in goats. However, its occurrence in the western region of the country, previously deemed a habitat for *Am. variegatum*, could be attributed to the effect of climate change on its distribution. Similarly, it has been identified in goats and cattle raised by the pastoral community of Kenya [59] and Sudan [55].

#### *Amblyomma cohaerens*

In Ethiopia, *Am. cohaerens* has been found scattered in many geographical locations. The highest burden of this tick in the western location, which is characteristically sub-humid, indicates the suitability of this region for its life-cycle. The occurrence of *Am. cohaerens* in South Sudan [55], a country that borders Ethiopia in the western region, indicates the likelihood of this tick crossing from one country to another. There has never been adequate evidence to show its host-specific nature. However, its highest burden in cattle might be explained by the presence of a huge cattle population in the region [60]. Meanwhile, its occurrence on camels in the southern, northern, and eastern regions not only illustrates its feeding on multiple hosts but also indicates its ability to thrive in arid and semi-arid agroecologies, where camels are raised. In certain African countries, this tick appears to infest African buffalo and elephants [61, 62], which further confirms its non-selective feeding.

#### *Hyalomma truncatum*

This tick can be found anywhere in the country. Its highest abundance in eastern arid and semi-arid agroecologies reveals its drought-resistant trait. Although it affects a wide range of domestic animals, the highest infestation rate in goats suggests the presence of much of the goat population raised in arid and semi-arid agroecologies where this tick predominates. It has been shown that this tick circulates in almost all southern African countries and some in eastern Africa, such as Kenya and Sudan [58, 61, 63]. It appears that the tick inevitably infests many domestic animals and wildlife in these regions.

#### *Hyalomma rufipes*

This hairy medium-sized tick generally occupies all agroecology zones of the country, but its highest abundance in lowland arid agroecology indicates its drought-tolerant trait. It is the most widely distributed tick among many *Hyalomma* species and often coexists with *Hy. truncatum.* The highest burden of this tick in goats further explains the concordance of its distribution with the natural lowland agroecology, where goats are allowed to freely graze in Ethiopia. It has been found to infest wild animals besides domestic livestock in several southern African countries and Kenya [58, 61]. This tick also exists in Sudan and the Republic of South Sudan [55], a neighboring country of Ethiopia in the northwestern region. Moreover, its highest abundance in the eastern region of Ethiopia suggests the possibility of this tick occurring in neighboring countries in the eastern region, such as Somalia.

#### *Hyalomma dromedarii*

Previously, this tick was thought to be a camel tick in many countries, as reflected in its name meaning “camel” and given that its distribution often coincided with camel-rearing areas. However, there is growing evidence that this tick has been infesting other domestic animals, such as cattle [64] and goats [65], which are reared together with camels in different countries. Similarly, in Ethiopia, although the highest burden of this tick infestation is in camels, available evidence shows that other livestock are also infested. The burden in camels shows coincident distribution with camel production areas, which are characteristically arid or semi-arid, where pastoralists herd camels together with other domestic animals, including cattle and goats. A study conducted in arid and semi-arid agroecologies in Pakistan [65] supports this argument. The occurrence of this tick on camels and other domestic animals in many Middle East Arab countries has been reviewed [64]. Moreover, evidence reveals that *Hy. dromedarii* infests camels and ruminants in arid and semi-arid agroecologies of East African countries such as Kenya, Sudan, and Somali [63, 66, 67]. Interestingly, the absence of this tick in the western region, including B-G of Ethiopia and South Sudan, a country that borders Ethiopia at a western location, is evidence of the unsuitability of the area for this tick.

#### *Hyalomma excavatum*, *Hyalomma impeltatum*, *Haemaphysalis leachi*, and *Rhipicephalus bergeoni*

In Ethiopia, *Hy. excavatum* and *Hy. impeltatum* are rarely reported ticks, unlike in other nations such as Middle East Arab nations including Sudan [64], where these ticks are a major vector and the most frequently encountered pest in domestic animals. In Pakistan [65] and many African countries [11], in addition to *Hy. excavatum*, *Hyalomma anatolicum* appears to be the most abundant tick. Nonetheless, these species have been hardly distinguished, and their distinct morphological features could not be established in Ethiopia. Consequently, with the exception of one study [54], all studies reported these two tick species as one tick, namely *Hy. anatolicum excavatum*. They have never been reported in Kenya despite these ticks being rarely observed in southern Ethiopia along the border with southern Kenya. The few reports of *Hy. impeltatum* in Kenya and Ethiopia at the border region suggest that this tick could simultaneously thrive in both countries or cross from one country to another. The report of *Ha. leachi* from dogs in a central town only indicates that dogs dwelling in urban areas were given greater attention when compared to dogs living in rural settings. This tick was also reported from dogs that visited the animal hospital at Jos, Plateau State, Nigeria [68]. *Rhipicephalus bergeoni*, previously reported from central and eastern Ethiopia, is now seldom reported or completely absent in almost all surveyed sites. The long-term climate impacts might have contributed to the disappearance of this tick from the region.

#### *Rhipicephalus pulchellus*

The distribution of *Rh. pulchellus* in arid and semi-arid lowlands of Ethiopia coincides with reporting that this tick is present mainly in the Rift Valley of the Horn of Africa. It infests all domestic animals in the country except cats. Its infestation in humans in Kenya [69] confirms its non-host-specific feeding behavior. The highest abundance of this tick in camels as observed in the current report implies that camel-rearing areas in Ethiopia, such as lowland arid and semi-arid agroecologies, favor its biology. Its occurrence on cattle, buffalos, and camels in Kenya, Uganda, and some Middle East Arab countries has also been reported [57, 64, 70].

#### *Rhipicephalus evertsi evertsi*

As with other *Rhipicephalus* species, *Rh. evertsi evertsi* thrives in almost all agroecological zones and geographical locations in Ethiopia. The wide geographical distribution of this tick observed in a neighboring country [55] supports the above argument. The observations of this tick on several domestic animals point to its non-host-specific feeding, although its abundance is often higher in large animals when compared with small animals. This is in line with the report from Kenya [57]. Considering its wide geographical coverage, tick-borne diseases (TBDs) associated with *Rh. evertsi evertsi* should be suspected in all geographical locations.

#### *Rhipicephalus* (Boophilus) *decoloratus*

*Rhipicephalus* (Boophilus) *decoloratus* is like *Rh. evertsi evertsi* in that it covers wide geographical locations and is presumed to exist in almost all agroecologies. Nonetheless, its higher burden in warm sub-humid agroecology indicates its importance in this area. A similar finding was reported in the warm humid forest zone of Cameroon [56]. Thus, strategic control measures against the tick and pathogens it transmits are warranted in the warm sub-humid agroecology of Ethiopia. It feeds on a wide range of hosts, including wildlife [71], and this may hasten the transmission of pathogens among livestock populations.

#### *Rhipicephalus sanguineus* s.l.

As in other parts of the world, *Rh. sanguineus* s.l. in Ethiopia is primarily associated with dogs but can infest other animals as well. The same observation was reported in Sudan, where *Rh. sanguineus* s.l. infests cattle [63]. The identification of this tick on dogs living with domestic ruminants in Egypt [72] is a good indication that this tick can attack any domestic animal living in close association with dogs. Besides, close intimacy of dogs and humans in urban and rural areas may increase the likelihood of accidental human infestations [73] thus may pose TBDs. The report of this tick only in central Ethiopia does not mean its absence in other locations but it does show tick studies in dogs were confined to major towns and cities that are near to central Ethiopia. Given its public health risk and veterinary importance, the author suggests studies on TBDs of humans and animals in areas where this tick prevails.

#### *Rhipicephalus pravus*

A significant abundance of *Rh. pravus* in the lowland arid and semi-arid agroecologies of Ethiopia justifies its drought-resistant nature. This allows its survival during the dry season, thus becoming endemic throughout the year in the environment. Infestation of this tick on cattle and other domestic animals suggests that it is not host-specific. The high burden of this tick next to *Rh. pulchellus* on camels reared in the lowland arid agroecological zone of Kenya [66] supports the above explanation. Moreover, the tick appears to harbor an agent of Q fever; thus, an epidemiological study about this zoonotic infection should be initiated in the lowland arid and semi-arid agroecologies to design control strategies.

#### *Rhipicephalus lunulatus*

Unlike other *Rhipicephalus* species identified in Ethiopia, *Rh. lunulatus* is seldom reported, found only in Oromia and southern Ethiopia. The absence of this tick in many surveyed regions and lack of data within the past 10–20 years remains a great concern. This might be linked with climate change that could have hampered the ecology and biology of the tick. In Nigeria, one study [74] identified only one *Rh. lunulatus* on cattle in sub-humid agroecology during the rainy season. However, the issue of seasonal impact on its abundance needs further investigation. Moreover, the pathogens they may transmit in livestock have never been identified. Hence, studies on the seasonal abundance and TBPs in this tick are highly encouraged.

#### *Rhipicephalus muhsamae*

As with other drought-resistant ticks, *Rh. muhsamae* thrives in the lowland arid and semi-arid agroecology of Ethiopia. The presence of this tick has been demonstrated in almost all regions in Sudan [55]. Nonetheless, unlike other *Rhipicephalus* species, its limited distribution only in Ethiopia and Sudan raises questions as to the specific factors contributing to its distribution.

#### *Rhipicephalus praetextatus*

*Rhipicephalus praetextatus* appears to coexist with many drought-resistant *Rhipicephalus* species in Ethiopia. Its geographical distribution in lowland semi-arid agroecology in Ethiopia accords with reports from neighboring countries, such as Kenya and Sudan [55, 57].

### Distribution of TBPs

#### *Anaplasma* species

To date, five *Anaplasma* species, namely *Anaplasma centrale*, *Anaplasma marginale*, *Anaplasma* sp. ‘Omatjenne,’ *Anaplasma phagocytophilum*, and *Anaplasma ovis*, along with other unidentified species [14], have been reported in different regions. Of the identified *Anaplasma* pathogens, almost all are endemic in the western region of Ethiopia, as many suitable tick vectors are present in this region. However, some of them, such as *A. centrale*, *A. marginale*, and *A.* sp. ‘Omatjenne,’ are found to infect livestock in central and southern areas of the country, suggesting the existence of common tick vectors for these pathogens in western, central, and southern geographical locations. Moreover, reports of *A. ovis* and *A. phagocytophilum* infections of livestock in western and central regions confirm the occurrence of their respective tick vectors in the areas. Teshale et al. [49] demonstrated that *Rh. evertsi evertsi* and *Rh*. (B.) *decoloratus* are vectors of *A. ovis*. In Kenya, this pathogen was identified in *Am. variegatum*, *Rh. pulchellus*, and *Am. gemma* [75], proving the involvement of multiple species of ticks. Thus, infection of small ruminants with *A. ovis* should be suspected in areas in which these ticks are prevalent. They are abundant in almost all geographical locations, including in western, southern, and central areas. Meanwhile, *Am. cohaerens*, which circulates in many areas including in western and central regions, was found to transmit *A. marginale* [72] and *A. phagocytophilum* [18], which is a zoonotic pathogen causing human granulocytic anaplasmosis. In Europe, however, this zoonotic infection is transmitted by *Ixodes ricinus* [76], confirming that TBPs can use different tick species depending on their distribution. The tick species implicated as vectors of *A. centrale* and *A.* sp. ‘Omatjenne’ could not be identified. From the perspective of controlling ticks and TBPs, the vectors for these pathogens should be determined.

#### *Ehrlichia* species

The causative agent of bovine ehrlichiosis*, Ehrlichia ruminantium*, and other unconfirmed species were detected in *Rh*. (B.) *decoloratus* in the southwestern region of Ethiopia. Bovine infection with this pathogen had been elucidated in the southwestern region of the country [14]. Interestingly, this pathogen appears to thrive in another tick species, *Am. gemma,* in the eastern Somali region [16]; nonetheless, absence of infection in cattle in this region may suggest its poor vector competence to *E. ruminantium.* In neighboring Sudan, *Am. variegatum* and *Am. lepidum* have been implicated as the major vectors [64], whereas *Am. gemma*, *Am. variegatum*, and *Rh. evertsi evertsi* were found to harbor *E. ruminantium* in Kenya [75]. This evidence implies that bovine ehrlichiosis can occur in the regions in which any of these ticks exist. A doubtful *Ehrlichia* species in *Rh.* (B.) *decoloratus* collected from the southwestern area could be *E. minasensis*, as infection of livestock with this agent was discovered in the same region [14].

#### *Rickettsia* species

*Rickettsia africae*, an agent for African tick bite fever (ATBF), and unconfirmed species have been reported from western and eastern regions. *Rhipicephalus* (B.) *decoloratus* and *Rh. evertsi evertsi* are the major vectors for *R. africae* in western locations [49], while *Am. gemma* was found to be a vector responsible for this pathogen in the eastern Somali region [16]. In Kenya, *Am. variegatum*, *Am. gemma*, and *Rh. evertsi evertsi* are implicated as vectors of *R. africae* [75, 77]. This shows that the distribution of agents for ATBF may coincide with the distribution of these ticks in Ethiopia. Nevertheless, reports of human cases are largely lacking, with only one case report in a patient who had a history of travel [78].

*Rickettsia aeschlimannii*, an agent of spotted fever group (SFG) rickettsiosis, was confirmed in three tick species, including *Hy. impeltatum*, *Hy. rufipes*, and *Hy. truncatum*, in eastern Somali using a molecular technique [50, 79]. Similarly, researchers have detected this pathogen in *Hy. truncatum*, *Hy. rufipes*, and *Rh. pulchellus* in Kenya [75]. In addition, many other *Hyalomma* species are implicated as vectors for *R. aeschlimannii* in Egypt and Algeria, suggesting potential vector competence of *Hyalomma* species. Thus, SFG rickettsiosis should be considered when diagnosing patients with a history of tick bites in regions where *Hyalomma* species are abundant. Furthermore, *Hyalomma* species are found to carry *Francisella*-like endosymbiont [53], which is a zoonotic pathogen that causes tularemia. However, despite the wide prevalence of these ticks, cases are seldom reported, and only in patients with a history of travel to endemic areas.

#### *Theileria* species

More than five molecularly confirmed *Theileria* species (Additional file 3: Fig. S3) are implicated as causative agents of theileriosis infections in the southwestern and northern regions [14, 51, 52]. Theileriosis is a health threat to livestock in many areas of the country, given a wide distribution of tick vectors. Fortunately, many *Theileria* species, including *Theileria mutans*, *Theileria velifera*, *Theileria ovis*, and *Theileria orientalis*, are mildly pathogenic and cannot result in overt clinical disease. *Rhipicephalus.* (B.) *decoloratus* and *Rh. evertsi evertsi* appear to harbor a mild form of *Theileria* pathogens. However, a pathogenic strain, *Theileria annulata*, that causes tropical theileriosis is endemic in the southern region of the country [52].

There have been many studies on TBPs in the western area, but the absence of *T. annulata* in this region is interesting. Its vector is not yet identified in the country; however, the occurrence of this pathogen in southern areas reveals the existence of a specific tick, which is deemed uncommon in western regions. In Sudan, researchers were able to identify *T. annulata* from *Hy. anatolicum* [80], which is a rarely reported tick in the western region of Ethiopia but frequently encountered species in southern Ethiopia. This shows that the tick *Hy. anatolicum* might be a potential vector for *T. annulata* in Ethiopia. Therefore, surveillance of tropical theileriosis should be considered in the area in which *Hy. anatolicum* circulates.

#### *Babesia* species

There have been controversial issues as regards the reporting of *Babesia* species in Ethiopia. Of the *Babesia* species, only *B. bigemina* seems to have been implicated as an agent of bovine babesiosis, as it was confirmed in cattle with a molecular technique [14]. On the other hand, one study demonstrated bovine babesiosis caused by *B. bovis* in the same area by microscopic examination [12]. The ticks *Rh.* (B.) *decoloratus*, *Rh.* (B.) *annulatus*, and *Rh.* (B.) *microplus* are thought to be responsible vectors for *B. bigemina* in many tropical countries, while only *Rh*. (B.) *annulatus* and *Rh.* (B.) *microplus* are major vectors for *B. bovis* in Latin America and other parts of the world [80, 81]. Therefore, the absence of *Rh.* (B.) *annulatus* and/or *Rh.* (B.) *microplus* in the southwestern region of Ethiopia and low sensitivity of microscopic examination for pathogen detection might have contributed to misleading positive diagnosis in the previous study. Reporting from Sudan confirms the presence of *B. bovis* and *B. bigemina* in *Rh.* (B.) *annulatus*, and the absence of *B. bovis* in *Rh.* (B.) *decoloratus* supports the argument that *Rh.* (B.) *decoloratus* does not transmit the agent *B. bovis.* In addition, *B. caballi* has been isolated from the tick species [35]. However, the infection status in equine populations has not yet been established; thus epidemiological investigation using the molecular method in all domestic livestock is of paramount importance.

#### *Coxiella burnetii*

*Coxiella burnetii*, a zoonotic pathogen that causes Q fever in humans, was detected from Ethiopia in hard ticks using molecular techniques [36, 82]. These studies show that multiple ticks contribute to the perpetuation of *C. burnetii* in the country. However, a significantly higher infection rate occurs in *Am. gemma* and *Rh. pulchellus* [82], suggesting its magnitude might be higher in lowland arid and semi-arid agroecologies, where these ticks are abundant*.* In addition, other *Amblyomma* species, including *Am. variegatum* and *Am. cohaerens*, have been found to carry the *C. burnetii* genotype*.* This highlights that *Am. gemma*, *Am. variegatum*, and *Am. cohaerens* seem to be the major vectors in southwestern Ethiopia given their high burden in this region. Moreover, the seropositivity of Q fever in humans and livestock in eastern Somali [83] and the southeastern Oromia region [84] reveals the importance of Q fever from both a public health and veterinary perspective.

## Conclusions

Ixodid ticks and TBPs pose significant health threats to livestock in Ethiopia. To date, ticks have been reported from all administrative regions of the country except Gambela and Afar. This highlights the need for future study on ticks and TBPs in these regions in general and the Gambela region in particular, as *Rh. appendiculatus*, a vector of East Coast fever, is prevalent in South Sudan, a country bordering Gambela, Ethiopia. TBPs have been reported from only a few administrative regions of Ethiopia, even though ticks are widely distributed in the country. Thus, the author encourages future studies to detect TBPs from ticks and livestock using molecular techniques to gain a clearer picture of TBDs and to aid in the design of control techniques. Individual tick distribution and abundance depend on the nature of agroecology and the presence of a suitable host. This helps identify the areas of lowest and highest risk for better management of genetically improved livestock breeds. Moreover, all ixodid ticks can infest a wide range of mammalian hosts, including humans; however, there has never been a report on ticks infesting wild animals in the country. Hence, the contribution of wild animals to the epidemiology of ixodid ticks and TBPs in the country should be established.

## Supplementary Information

**Additional file 1: Fig. S1.** Abundance of tick species on different domestic animals in Ethiopia.

**Additional file 2: Fig. S2.** Abundance of tick species in various geographic locations of Ethiopia.

**Additional file 3: Fig. S3.** Abundance of tick species in different agroecological zones of study areas of Ethiopia.

## Data Availability

All data generated or analyzed during this study are included in this article and its supplementary information files.

## Abbreviations

TBPs: Tick-borne pathogens
SNNPR: Southern Nations, Nationalities, and Peoples' Region
TBDs: Tick-borne diseases
ATBF: African tick bite fever
SFG: Spotted fever group
B-G: Benishangul-Gumuz region
