# Supplementary figures and images for: Geographical distribution of ixodid ticks and tick-borne pathogens of domestic animals in Ethiopia: a systematic review
Source: Parasit Vectors. 2022 Mar 28;15:108. doi: 10.1186/s13071-022-05221-x (PMC8961985; doi:10.1186/s13071-022-05221-x)

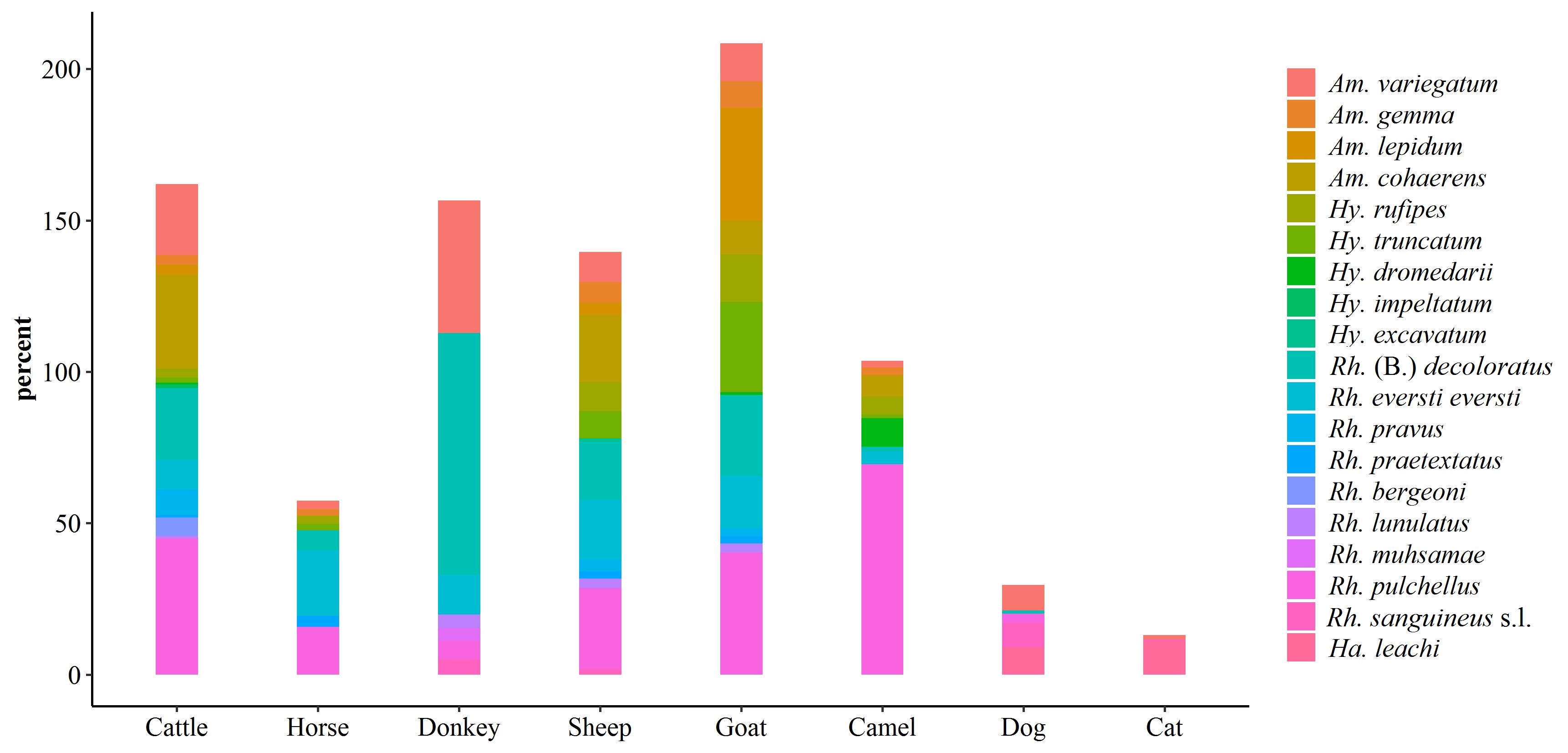

Supplement: Supplementary file 1 — Additional file 1: Fig. S1. Abundance of tick species on different domestic animals in Ethiopia. [file 13071_2022_5221_MOESM1_ESM.jpg]

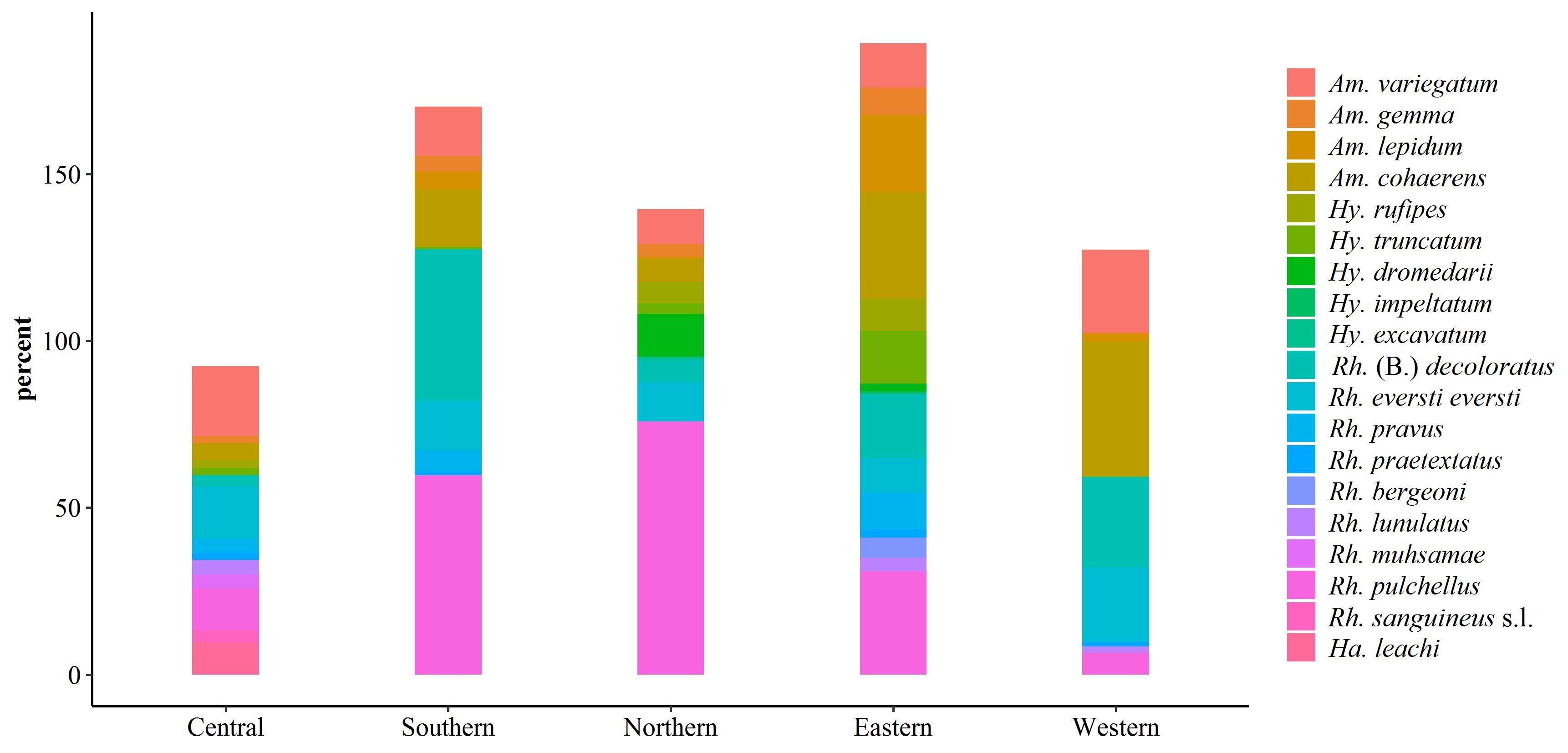

Supplement: Supplementary file 2 — Additional file 2: Fig. S2. Abundance of tick species in various geographic locations of Ethiopia. [file 13071_2022_5221_MOESM2_ESM.jpg]

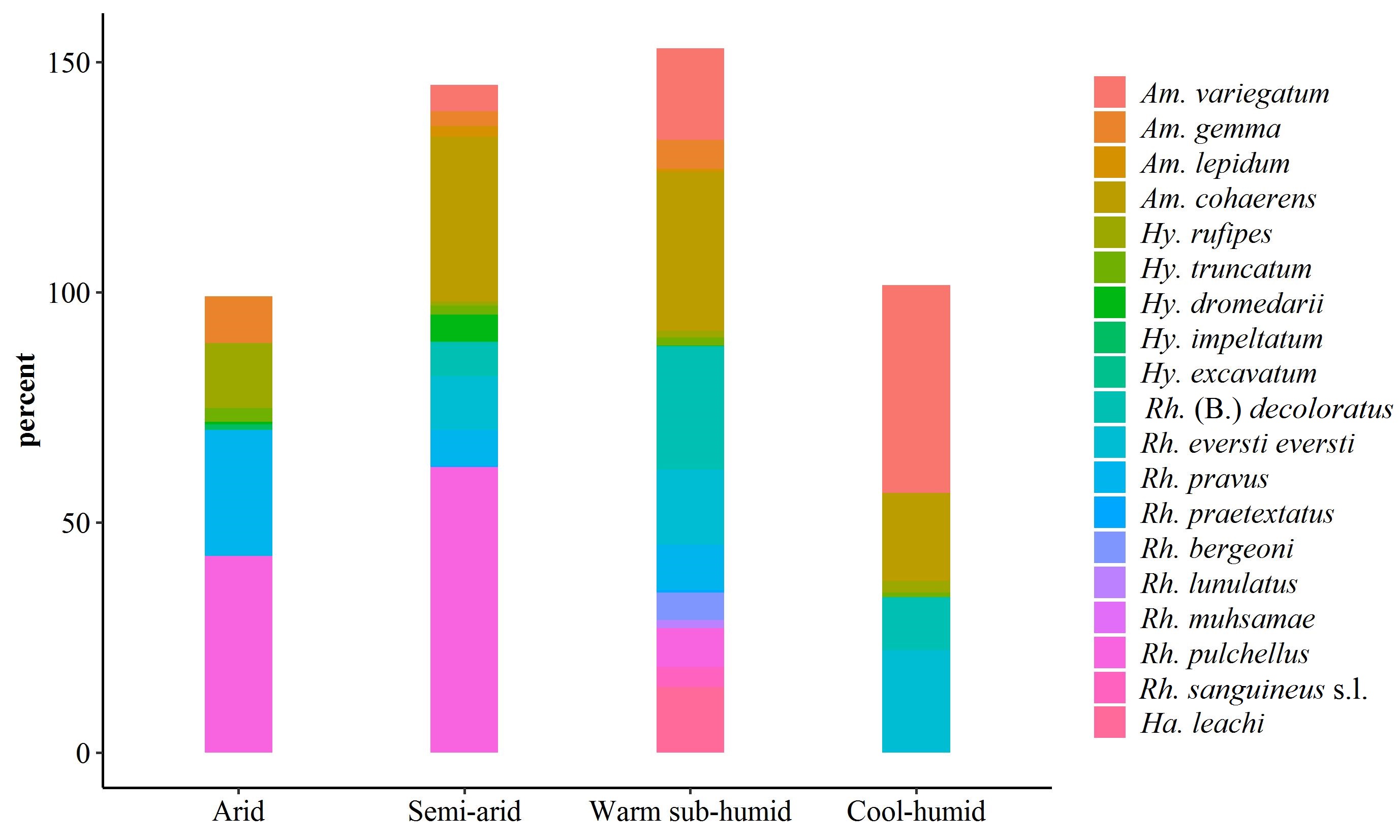

Supplement: Supplementary file 3 — Additional file 3: Fig. S3. Abundance of tick species in different agroecological zones of study areas of Ethiopia. [file 13071_2022_5221_MOESM3_ESM.jpg]
